# Supplementary material for: Anti-progestin therapy targets hallmarks of breast cancer risk
Source: Nature. 2025 Nov 5;648(8094):736–45. doi: 10.1038/s41586-025-09684-7 (PMC12711567; doi:10.1038/s41586-025-09684-7)
Supplement: Supplementary file 1 — Breast Cancer-Anti-Progestin Prevention Study 1 (BC-APPS1) clinical study protocol. [file 41586_2025_9684_MOESM1_ESM.pdf]

nature portfolio

https://doi.org/10.1038/s41586-025-09684-7

Supplementary information

Anti-progestin therapy targets hallmarks of breast cancer risk

In the format provided by the authors and unedited

Short Title: Breast Cancer - Anti-Progestin Prevention Study 1

(BC-APPS1)

Full Title: A pilot prevention study of the effects of the anti-progestin Ulipristal Acetate (UA) on surrogate markers of breast cancer risk

Eudract Number:
2015-001587-19

Sponsor's reference number:
UHSM0315

Version No and Date: Version 2.0 21/12/2018

Confidentiality Statement

This document contains confidential information that must not be disclosed to anyone other than the Sponsor, the Investigator Team, the Sponsoring Trust's R&D Office (or a regulatory authorities, and members of the Health Research Authority or Local Research Network).

Sponsor: Manchester University NHS Foundation Trust

Education and Research Centre,

Southmoor Road,

Manchester,

M23 9LT

Tel: 0161 291 5775

Fax: 0161 291 5771

Email: faye.o'keeffe@manchester.ac.uk

Funder: Breast Cancer Now

Fifth floor, Ibex House,

42-47 Minories,

London

EC3N 1DY

Tel: 020 749 4108

www.breastcancernow.org

Chief Investigator: Dr Sacha Howell

School of Molecular & Clinical Cancer Sciences

Faculty of Biology, Medicine and Health

University of Manchester,

The Christie NHS Foundation Trust, department of Medical Oncology

Wilmslow Road,

Manchester,

M20 4BX

Tel: 0161 446 8347

Fax: 0161 446 3299

Email: Sacha.Howell@Christie.NHS.UK

Signature page

The approved protocol should be signed by author(s) and/or person(s) authorised to sign the protocol

The undersigned confirm that the following protocol has been agreed and accepted and that the Chief Investigator agrees to conduct the trial in compliance with the approved protocol and will adhere to the principles outlined in the Medicines for Human Use (Clinical Trials) Regulations 2004 (SI 2004/1031), amended regulations (SI 2006/1928) and any subsequent amendments of the clinical trial regulations, GCP guidelines, the Sponsor's SOPs, and other regulatory requirements as amended.

I agree to ensure that the confidential information contained in this document will not be used for any other purpose other than the evaluation or conduct of the clinical investigation without the prior written consent of the Sponsor

I also confirm that I will make the findings of the study publically available through publication or other dissemination tools without any unnecessary delay and that an honest accurate and transparent account of the study will be given; and that any discrepancies from the study as planned in this protocol will be explained.

For and on behalf of the Study Sponsor:

Signature: .... Date: ....../....../......

Name (please print):...

Position: ....

Chief Investigator:

Signature: .... Date: ....../....../......

Name: (please print):...

Trial Management Staff

**Role:**	**Name:**	**Contact:**
Trial Manager	Faiza Idries	Telephone: 01612914408<br>Faiza.Idries@MFT.NHS.UK
Chief investigator	Dr Sacha Howell	Telephone: 0161446 8347<br>Sacha.Howell@Christie.NHS.UK
Co-Investigator	Prof Anthony Howell	Tony.Howell@ics.Manchester.ac.uk
Co-Investigator	Prof D Gareth Evans	Gareth.Evans@cmft.nhs.uk
Sponsor representative	Dr Karen Hampson/Faye O'Keefe/Sian Hanison	karen.hampson@manchester.ac.uk<br>faye.o'keeffe@manchester.ac.uk<br>sian.hanison@manchester.ac.uk
Imaging specialists	Dr Anthony Maxwell<br>Dr Yit Lim	Anthony.Maxwell@MFT.NHS.UK<br>Yit.Lim@MFT.NHS.UK
Breast Biology lab	Dr Robert Clarke	Robert.Clarke@Manchester.ac.uk
Breast Stroma lab	Prof Charles Streuli	Charles.Streuli@Manchester.ac.uk
Bioinformatics support	Dr Andrew Simms	Andrew.Simms@Edinburgh

Amendment History

Superseded Version	Reason for Change
**1.6**	Added trial reference numbers.<br>Change of Sponsor representative.<br>Additional information added relating to the Investigational Medicinal Product<br>Altered eligibility criteria regarding blood tests and biopsy review by pathologist<br>Addition of urine pregnancy tests at visits.<br>Addition of patient weight at each visit<br>Clarification of vaginal bleeding on study that would lead to discontinuation of study medication<br>Amended visit window for screening visit<br>Amended exclusion criteria to enable participants not having an MRI to continue in the trial
**1.7**	"Telephone call on Monday after predicted OM date" visit has been amended to "Telephone call to patient regarding histology results and onset of menses"; there has also been further clarification of when patient should start medication added and further activity to be completed during this visit added.
**1.8**	-Page 1 – Breast Cancer Campaign logo has been changed to Breast Cancer Now logo to reflect the merger of the two charities Breast Cancer Campaign and Breakthrough Breast Cancer.<br>•Sponsor has been changed from "University of Manchester NHS Foundation Trust" to "Manchester Univeristy NHS Foundation Trust"<br>•Page 4- Email addresses for Dr Anthony Maxwell, Dr Yit Lim and Faiza Idries have been amended.<br>•Page 19 **section 5.4.3 Drug supply and accountability -** the last sentence of the second paragraph will be changed from "The participant will be asked to bring all unused medication to the clinic at each visit where it will be returned to pharmacy" to "The participant will be asked to bring all unused medication to the clinic at each visit where it will be checked for compliance and, at the end of treatment, returned to pharmacy."<br>•Page22 **section 6.2. Exclusion Criteria –** Addition of the following criteria:-Previous/current diagnosis of hepatitis -Diagnosis of Cirrhosis of the liver<br>•Page 24 **Screening visit (day – 7 ± 4 days EOM) -** Addition of measuring patient's height at screening visit.<br>–Deletion of measuring AST at screening visit as this is not a value that would impact the participants' eligibility to take the Ulipristal Acetate. This has therefore been deleted from the screening visit section and Appendix 5 (ALT will continue to be monitored).<br>•Page 25 **VAB visit (day – 7 ± 4 days EOM to OM)**<br>-Bullet point number 5 has been changed from "A 28 day supply of 5mg UA will be supplied to the participant who will be instructed to begin taking them on the first Monday after their next menstrual period begins" to "3x 28 day pack supply of 5mg UA will be supplied to the participant who will be instructed to wait for a telephone call from the study team before starting the medication. Participant will also be given a study card with the medication supply"<br>-Bullet point number 6 has been changed from "The patient will undergo

	an ECG of the breast to produce a breast electrical impedance measurement" to "The patient will undergo breast electrical impedance measurement"<br>-Bullet point number 7 has been changed from "The patient will undergo the VAB" to "The patient will undergo the VAB of one breast"<br>•Page 26 **Telephone call to patient regarding histology results and onset of menses cont.**<br>-Bullet point 4 has been changed from "If the participant has started their period, and there is no evidence of cancer or DCIS on the breast biopsy, they will be instructed to start study medication" to "If the participant has started their period, and there is no evidence of cancer or DCIS on the breast biopsy, they will be instructed when to start study medication . This will be within 1 week of onset of menstruation (OM), in line with the UA SPC."<br>-Bullet point 5 has been changed from "If the participant has not started their period then they will be instructed to commence study medication (UA) at the onset of menstruation (OM) and to contact the research team on the next working day (M-F) after that date. If the participant has not phoned by a maximum of 5 days past her EOM then the research team will contact her to ask if she has started her period. If so then she will be asked the date of OM and the date she started study medication and if not she will be advised again to start study medication with OM and to contact the research team again as above. In line with the UA SPC treatment should be started within 1 week of OM." to "If the participant has not started their period then they will be instructed to contact the research team at the OM or on the next working day (M-F) and they will be advised when to commence study medication (UA). If the participant has not phoned by a maximum of 5 days past her EOM then the research team will contact her to ask if she has started her period. If so then she will be asked the date of OM and will be advised when to start UA. If the participant has still not started her period she will be asked to contact the research team again as above. In line with the UA SPC treatment should be started within 1 week of OM."<br>•Page 26 **Visit 1 Day 29 + 4 / - 7 days (week 5)-** blood samples taken for LFT of which bilirubin, ALT, ALP, LDH will be measured has been added<br>- Deletion of bullet point 7 "receive a 28 day supply of 5mg UA and be instructed to begin taking them as soon as their previous supply finishes"<br>•Page 27 **Visit 2 Day 57 + 4 / - 7 days (week 9)-** blood samples taken for LFT of which bilirubin, ALT, ALP, LDH will be measured has been added<br>-Deletion of bullet point 7 "receive a 28 day supply of 5mg UA and will be instructed to begin taking them as soon as their other previous supply finishes"<br>•Page 28 **Visit 3 Day 78-85 (week 12) –** bullet point 2 has been changed to specify the LFTs that will be measured - bilirubin, ALT, ALP and LDH

	•Page 28 **VAB 2 visit/ Prophylactic Mastectomy visit Day 78-85**<br>-Bullet point number 3 has been changed from "The patient will undergo an ECG of the breast to produce a breast electrical impedance measurement" to "The patient will undergo breast electrical impedance measurement"<br>•Page 28 **VAB 2 visit/ Prophylactic Mastectomy visit Day 78-85 cont.**<br>Bullet point number 5 has been changed from "undergo the VAB/ Prophylactic Mastectomy" to "undergo the VAB of the breast that was not biopsied at baseline or Prophylactic Mastectomy"<br>•Page 28 Should a participant experience treatment emergent clinical symptoms or signs of liver disease whilst participating in the study they will be advised to contact the research team as soon as possible for assessment which will include repeat LFTs. Therefore, assessments of this nature could be conducted outside of the scheduled study visits.<br>• **Retrospective collection of weight and height**<br>-Participants who have completed the study or did not have their height recorded at their baseline appointment will be contacted by a member of the team and asked for their height, this will then be recorded in the participant's notes.<br>-Where weight was not collected, the weight used to calculate the dose of contrast at MRI will be used.<br>•Page 28 **Visit 4 Day 113 ± 4 days (week 17) -** blood samples taken for LFT of which bilirubin, ALT, ALP, LDH will be measured has been added<br>•Page 44 **section 14 Finance** – Breast Cancer Campaign address has been changed to Breast Cancer Now address to reflect the merger of the two charities Breast Cancer Campaign and Breakthrough Breast Cancer.<br>•Page 60 **Appendix 5**:<br>-Addition of "Height recorded" to table<br>-Addition of the measuring LFTs at weeks 5, 9 and 17 has been added<br>- The LFTs measured have been amended from " LFT (for ALT, AST, ALP, LDH)/" to " LFT (for bilirubin, ALT, ALP and LDH)".<br>-Deletion of measuring AST at screening visit<br>-Deletion of measuring AST at Visit 3 Day 78-85 (week 12)<br>-"ECG of the Breast" has been changed to "breast electrical Impedance"<br>-Deletion of the week 5 and week 9 dispenses from table<br>-Deletion of the week 5 and week 9 'patient to start 28 day supply of dispensed UA' checks from the table<br>-"Dispense ulipristal acetate 5mg daily x 28 days" changed to "Dispense ulipristal acetate 5mg daily: 3 x 28 days packs"<br>-"Patient to start 28 day supply of dispensed UA" changed to "Patient to start supply of dispensed UA"
**1.9**	•**Section added - 5.6.4 ALT** If at any point when the ALT is measured it is found to be more than three times the upper limit of normal, UA must be permanently discontinued.

	•**Section 6.2 Exclusion criteria** has been changed from "Pregnant or planning for pregnancy in the next 6 months. Pregnancy must be excluded with serum βhCG <5nmol during screening" to "Pregnant or planning for pregnancy in the next 6 months. Pregnancy must be excluded with serum βhCG".<br>•**Section 7.1 Screening visit (day – 7 ± 4 days EOM) – Bullet point 9** has been changed from "have their eligibility for MRI scans assessed (contraindications to MRI, such as intracranial aneurysm clips, implanted electrical devices and intra-ocular metallic foreign bodies will exclude participants from undergoing an MRI but they can continue with all other trial procedures" to "have their eligibility for MRI scans assessed (contraindications to MRI, such as intracranial aneurysm clips, implanted electrical devices and intra-ocular metallic foreign bodies will exclude participants from undergoing an MRI but they can continue with all other trial procedures. In addition, should a participant not fulfil the size/weight requirements of the scanner or if the scanner breaks down resulting in a participant not having an MRI scan they can continue with other trial procedures)."<br>•**Section 7.1 Visit details - MRI visit (day – 7 ± 4 days EOM to OM) –** changed from "If a participant is unable to tolerate the MRI scan, for example due to claustrophobia, she may continue on study and undergo all other trial procedures." to "If a participant is unable to tolerate the MRI scan, for example due to claustrophobia, she may continue on study and undergo all other trial procedures. In addition, should a participant not fulfil the size/weight requirements of the scanner or if the scanner breaks down resulting in a participant not having an MRI scan they can continue with other trial procedures."<br>•**Visit 1 Day 29 + 4 / - 7 days (week 5) –** the following has been added "If ALT is more than three times the upper limit of normal, UA must be permanently discontinued."<br>•**Visit 2 Day 57 + 4 / - 7 days (week 9) -** the following has been added "If ALT is more than three times the upper limit of normal, UA must be permanently discontinued."<br>•**Visit 3 Day 78-85 (week 12) -** the following has been added "If ALT is more than three times the upper limit of normal, UA must be permanently discontinued." In addition "have their samples prepared and stored as described in section xx" changed to "have their samples prepared and stored as described in sections 8 and 9."<br>•**Appendix 5: Summary of trial procedures –** changed from "*MRI to be performed before VAB. Contraindications to MRI, such as intracranial aneurysm clips, implanted electrical devices and intra-ocular metallic foreign bodies, will exclude participants from undergoing an MRI." to "*MRI to be performed before VAB. Contraindications to MRI, such as intracranial aneurysm clips, implanted electrical devices and intra-ocular metallic foreign bodies, will exclude participants from undergoing an MRI. In addition, should a participant not fulfil the size/weight requirements of the scanner or if the scanner breaks down resulting in a participant not having an MRI scan they can continue with other trial procedures."

Abbreviations

AFM	Atomic Force Microscopy
AI	Aromatase Inhibitor
ALT	Alanine aminotransferase
ALP	Alkaline phosphatase
APTT	activated Partial Thromboplastin Time
AST	Aspartate transaminase
ARFI	Acoustic Radiation Force Imaging
βhcg	Beta Human chorionic gonadotropin
BC	Breast Cancer
BPE	Background Parenchymal Enhancement
BRCA 1/2	Breast Cancer Association genes 1/2
CE-MRI	Contrast Enhanced MRI
CRF	Case Report Form
DMEM	Dulbecco's Modified Eagles Medium
E	Estrogen
eGRF	estimated glomerular filtration rate
EOM	Estimated onset of menstruation
FBC	Full Blood Count
FGV	Fibroglandular Volume
GCP	Good Clinical Practice
Hb	Haemoglobin
HRT	Hormone Replacement Therapy
IMP	Investigational Medicinal Product
LDH	Lactate dehdrogenase
LFT	Liver Function Tests
MD	Mammographic Density

MDT	Multi-Diciplinary Team
mg	Milligrams
MRI	Magnetic Resonance Imaging
OM	Onsent of menstruation
P	Progesterone
PgR	Progesterone Receptor
PRA/B	Progesterone Receptor A/B isoforms
PT	Prothrombin time
RANK/L	Receptor activator of Nuclear Factor Kappa B/ Ligand
SERM	Selective Estrogen Receptor Modulator
TFT	Thyroid function tests
TSH	Thyroid-stimulating hormone
T4	Thyroxine
UA	Ulipristal Acetate
U & E	Urea and Electrolytes
USS	Ultrasound scan
VAB	Vacuum Assisted Biopsy

Contents

TITLE PAGESHORT TITLE: BREAST CANCER - ANTI-PROGESTIN PREVENTION STUDY 1 (BC-APPS1) 1

CI DETAILS 2

SIGNATURE PAGE 3

TRIAL MANAGEMENT STAFF 4

AMENDMENT HISTORY 5

ABBREVIATIONS 8

1. INTRODUCTION 13

1.1. RATIONALE 13

2. BACKGROUND 13

2.1. PRECLINICAL MODELS 13
2.2. PROGESTERONE AND THE HUMAN BREAST 13
2.3. PROGESTERONE AND RADIOLOGIC BREAST DENSITY 14
2.4. PROGESTERONE AND MAMMARY STROMA 14
2.5. ANTIPROGESTINS 15

3. HYPOTHESIS AND AIMS 16

4. TRIAL SUMMARY 16

5. TRIAL MEDICATION 18

5.1. INVESTIGATIONAL MEDICINAL PRODUCT 18
5.2. LEGAL STATUS OF THE DRUG 18
5.3 REFERENCE SAFETY INFORMATION AND KNOWN DRUG REACTIONS 18
5.3.1 Reference safety information 18
5.3.2 Known drug reactions and interation with other therapies 18
5.4. Drug storage, supply and labelling 19
5.4.1 Dosing regimen 19
5.4.2 Drug storage 19
5.4.3 Drug supply and accountability 19
5.4.4 Preparation and labelling of Investigational Medicinal Product 19
5.5 Subject Compliance 20
5.6 Trial Restrictions 20
5.6.1 Acceptable methods of contraception 20
5.6.2 Non-acceptable methods of contraception 20
5.6.3 Pregnancy/genial bleeding 20
5.7 Concomitant Medication 21

6. PARTICIPANT RECRUITMENT AND ELIGIBILITY 21

6.1. INCLUSION CRITERIA 21
6.2. EXCLUSION CRITERIA: 22
6.3 WITHDRAWAL OF SUBJECTS 23

7. VISIT DETAILS AND METHODOLOGY 24

7.1. VISIT DETAILS 24
8. METHODOLOGY 29
8.1. TIIMING OF STUDY ENTRY BY LUTEAL PHASE PROGESTERONE 29
8.2. **BIOPSY AND SAMPLE PROCESSING PROTOCOL** 29

8.2.1 Vacuum assisted biopsy (VAB)....29
8.2.2 Prophylactic mastectomy samples....29
8.3. RADIOLOGUCAL ASSESSMENTS....30
8.4. ENDPOINTS....30
8.4.1 Primary endpoints....30
8.4.2 Secondary endpoints....30

8.5. **Exploratory endpoints**....31

9. **Specific protocols for endpoint analyses**

9.1. KI67 ANALYSIS....31
9.2 CLONOGENIC ASSAYS....32
9.3 Morphological analysis, tissue stiffness and collagen analyses.... 32
9.4 Magnetic resonance Imaging.... 33
9.5 Exploratory analyses.... 33

10. STATISTICAL CONSIDERATIONS....34

11. TRIAL RISK CATEGORY, POTENTIAL BENEFITS AND RISKS FOR PARTICIPATING PATIENTS....35

11.1 BENEFITS....35
11.2 RISKS....36

12. FORMS AND PROCEDURES FOR COLLECTING DATA....36

12.1 CASE REPORTS FORMS....36
12.2 DATA FLOW....37
12.3 WHAT TO RECORD IN THE PARTICIPANT FAMILY HISTORY MEDICAL RECORDS....38

13. SAFETY REPORTING AND PHARMACOVIGILANCE....38

13.1. RECORDING AND REPORTING OF SAES AND SUSARS....40
13.2. RESPONSIBILITY FOR SAFETY REPORTING....41
13.3. OUT OF HOURS CONTACT ARRANGEMENTS....43
13.4. THE TYPE AND DURATION OF THE FOLLOW-UP OF SUBJECTS AFTER ADVERSE EVENTS....43
13.5. REPORTING URGENT SAFETY MEASURES....43
13.6. NOTIFICATION OF DEATHS....43
13.7. PREGNANCY REPORTING....43
13.8. OVERDOSE....43
13.9. TREATMENT STOPPING RULES....44
13.10. DEVELOPMENT SAFETY UPDATE REPORTS....44
13.11. SIGNAL REVIEW AND TREND ANALYSIS....44
13.11.1 Safety signal review.... 44
13.11.2 Trend analysis of adverse events.... 44

14. FINANCE....44

15. PUBLICATION AND DISSEMINATION....45

16. TRIAL GOVERNANCE....45

16.1. TRIAL MANAGEMENT GROUP....45

17. CONFIDENTIALITY....45

18. QUALITY CONTROL **46**
19. PROTOCOL COMPLIANCE **46**
20. AMENDMENTS **47**
21. INSURANCE/INDEMNITY **47**
22. REFERENCES **48**

APPENDIX 1: INHIBITORS/INDUCERS OF CYP3A4 NOT TO BE TAKEN WITH UA **53**
APPENDIX 2: DATA ENTRY INTO EXCEL & STATISTICAL SOFTWARE PROGRAMMES & QC CHECKS **55**
APPENDIX 3: A GUIDE TO PERFORMING CAUSALITY ASSESSMENTS **58**
APPENDIX 4: SAE REPORTING PROCEDURES **59**
APPENDIX 5: SUMMARY OF TRIAL PROCEDURES **60**

1. Introduction

1.1. Rationale

1.4 million women worldwide are diagnosed with invasive BC each year and over a third die from their disease. Uptake and adherence to licensed chemo-preventative agents, tamoxifen and raloxifene, is low due in part to their adverse toxicity profiles. There is an urgent need for effective, well tolerated and safe breast cancer chemo-preventative agents. Endogenous progesterone induces proliferation of the normal mammary stem/progenitor cell population and exogenous progesterone is well known to increases the risk of postmenopausal breast cancer. Taken together these data suggest antagonism of PgR signaling may be a fruitful approach in the prevention of BC. Ulipristal acetate (UA) is a well-tolerated anti-progestin already licensed for the treatment of benign uterine fibroids. This project will, for the first time, determine the effects of the PgR antagonist UA on the normal breast in women at increased risk of BC and correlate molecular with imaging (MRI) effects.

2. Background

2.1. Preclinical models

In preclinical studies, progesterone is vital for both pubertal side branching and lobular alveolar development of the mammary gland during pregnancy (Lydon et al., 1995; Brisken et al., 1998; Mulac-Jericevic et al., 2003). Both in the intact mouse mammary gland and normal human breast explants lobular alveolar development results from progesterone induced expansion of the mammary stem cell (MaSC)/progenitor pool (Asselin-Labat 2010; Joshi 2010; Gonzalez-Suarez 2010). Mechanistically, this expansion is mediated through paracrine proliferative signals including RANKL and Wnt4a, secreted from progesterone receptor (PgR) positive sensor cells and acting on PgR negative MaSC/progenitors (Graham 2009, Joshi 2010, Gonzalez-Suarez 2010, Obr 2012). In multiple rodent models deletion or inhibition of PgR or the RANK/RANKL pathway results in significant reduction in mammary carcinogenesis (Schramek 2010, Gonzalez-Suarez 2010, Lydon 1999, Poole 2006).

2.2. Progesterone and the human breast

In the normal human breast the two PgR isoforms (PRA and PRB) are co-expressed at comparable levels. Relative loss of PRB is seen with the development of atypia or malignancy and in women with germline mutations in BRCA1 or 2 (Mote 2002). Women with such mutations have double the serum progesterone levels compared to age matched wild-type controls (Widschwendter 2013). The combination of estrogen and progestin as hormone replacement therapy (E+P HRT) significantly increases BC incidence and mortality whereas estrogen alone (E-HRT) does not (Anderson 2004; Chlebowski 2003; Chlebowski 2010; Beral 2003). In premenopausal women breast epithelial cell proliferation is highest in the progesterone dominant luteal phase of the menstrual cycle and can be reduced by the antiprogestin mifepristone, although this drug has now been discontinued due its hepatic toxicity (Engman 2008; Potten C 1988; Pike 1993; Navarrete 2005).

2.3. Progesterone and radiologic breast density

Mammographic density (MD) is one of the strongest risk factors for BC, with women of highest MD having a BC risk 4-6 fold higher than those with the least dense breasts (Assi 2011). Several studies have shown small increases in MD in the luteal vs follicular phase of the menstrual cycle (Buist 2006; Ursin 2001; Hovhannisyan 2009, Morrow 2010). Randomised data demonstrate a dose and serum progesterone level dependent increase in MD with combined E+P HRT but not E-HRT (Couto 2012; Greendale 2003; Lee 2012; McTiernan 2005; McTiernan 2009). Anti-estrogens (SERMs, AIs and GnRH analogues) only reduce MD significantly in premenopausal women (Cigler 2011; Cuzick 2004; Cuzick 2011, Pearman 2010) suggesting that an indirect effect of anti-estrogens, such as down regulation of progesterone signalling, may be responsible for this effect.

Contrast enhanced magnetic resonance imaging (CE-MRI) may offer a more detailed insight into breast biology. MRI measurements of fibroglandular volume (MRI-FGV) correlate well with volumetric MD and BC risk (Wang 2013; King 2012) and may be more sensitive to small changes. The enhancement of normal glandular breast tissue following contrast injection (background parenchymal enhancement - BPE) also correlates with BC risk but is independent of MD (King 2011). BPE is reduced by SERMS and AIs in pre and postmenopausal women (reviewed in Pike 2013). In addition, several studies have recently demonstrated increased BPE in the luteal vs follicular phase of the menstrual cycle (Amarosa 2013; Kajihara 2013, Scaranelo 2013). These data suggest that BPE and MRI-FGV assessment through CE-MRI may offer significantly more predictive capacity than MD in the assessment of novel BC preventative agents.

Potential changes in tissue stiffness as measured by ultrasound elastography with acoustic radiation force imaging (ARFI) will also be explored and the findings will be correlated with the mammographic and MRI data. There is relatively little published on the stiffness of normal breast tissue and the changes in stiffness in response to hormonal changes (Golatta 2013, Jud 2012, Rzymski 2011). Potentially ARFI could provide an easy and readily available modality to measure the response of the breast to chemopreventive agents.

2.4. Progesterone and Mammary Stroma

Although perhaps counter-intuitive, dense vs non-dense areas of the breast have increased expression of matrix metaloproteases (MMP) and proteoglycans but reduced TGF-β (Guo 2003; Alowami 2003; Yang 2010). This phenotype is replicated in PR-A transgenic mice and can be reversed with mifepristone treatment (Simian M 2009; Ewan KB 2002). Expression of certain MMPs is higher in the luteal vs follicular phase in the normal breast and significant variation in proteoglycans through the menstrual cycle has previously been demonstrated (Ferguson 1997; Pardo 2014). In addition, the stromal:epithelial ratio in dense areas is 15:1 and the major component of the stroma is collagen (Ghosh 2012; Li 2005). We have recently demonstrated that fibrillar collagen abundance correlates highly significantly (R^2=0.87) with MD assessed using Volpara™ volumetric analysis (figure 1). Preclinical data demonstrate that it is collagen organisation and not abundance that is important in rat carcinogenesis (Maller 2013) and it is vital,

therefore, that both assessment of radiological density and stromal mechanical function at the cellular length scales are incorporated into this study.

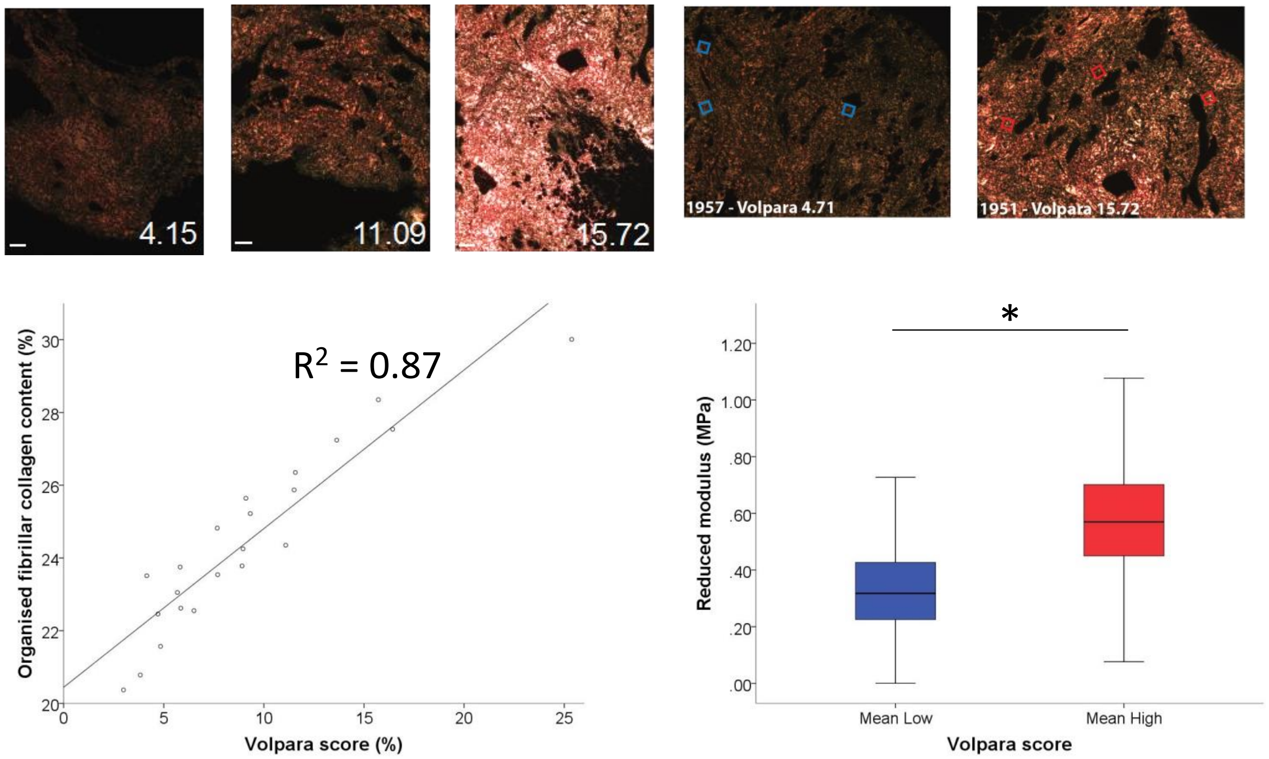

Figure 1. Mammographic density is positively correlated with organised fibrillar collagen content and with local micro-mechanical stiffness. Non-cancerous breast tissues samples from regions of high mammographic density were obtained post mastectomy from 30 women aged 55-67. A) Fibrillar collagen content as quantified by polarised light microscopy of picrosirius red stained tissue sections was correlated with global mammographic density as measured by Volpara scoring. B) Micro-mechanical stiffness (reduced modulus as quantified by atomic force microscopy) was significantly lower in individuals with low (<5) compared with high (>15) Volpara score ($p<0.01$, t test). These observations suggest that high mammographic density is associated with increased stiffness at cellular length scales.

2.5. **Antiprogestins**

PgR antagonists can be divided into three subtypes based on their effects on PgR phosphorylation and DNA binding. Type I PgR antagonists (eg Onapristone) induce minimal PgR phosphorylation and do not induce DNA binding. In contrast type II and III PgR antagonists induce both PgR phosphorylation and DNA binding. Type III antagonists such as lonaprisan are antagonists, even in the presence of cAMP, in contrast to type II antagonists such as mifepristone and UA, which show some agonist activity under such conditions. Onapristone and mifepristone have shown encouraging anti-BC activity in contrast to lonaprisan which was ineffective. Mifepristone was also shown to reduce normal breast proliferation, however, development as a BC treatment ceased due to hepatic toxicity which was likely mediated by non-specific activation of the glucocorticoid receptor (Engman 2008; Klijn 2000). In contrast UA 5mg (Esmya™), licensed for the treatment of uterine fibroids, shows >10 fold less anti-glucocorticoid activity than mifepristone (Attardi 2002), no additional toxicity over placebo, significant reduction in

toxicity compared with a GnRH agonist (Donnez 2012a and b) and no carcinogenicity or significant hepatotoxicity in rodent studies (Pohl 2013). Thus UA is an ideal candidate antiprogestin to test in this study.

3. **Hypothesis and aims**

The hypothesis to be tested is that modulating progesterone receptor signalling by UA reduces proliferation in luminal epithelial cells, alters the stromal composition, and reduces radiological density in the normal breast tissue of women at increased risk of BC. The outcome of this work will provide new data on predictive biomarkers, and potential treatments to reduce BC incidence in those at increased risk.

The aims of the study are therefore:

- To determine the effects of the antiprogestin ulipristal acetate (UA) on the epithelial and stromal compartments of the normal breast in women at increased risk of breast cancer (BC) and to relate these effects to quantitative changes on multiparametric magnetic resonance imaging (MRI) and ultrasound elastography.
- To define predictive imaging biomarkers for subsequent testing in randomised prevention trials of antiprogestins.

4. **Trial Summary**

A summary of trial procedures is provided in Appendix 4.

30 premenopausal women at moderate to high risk of BC (NICE guidelines) will be recruited from family history clinics in a similar manner to previous successful biopsy and dietary intervention studies (Ong 2009; Evans 2010), using mailshot and clinic visit recruitment. Subjects will be confirmed to be in an ovulatory menstrual cycle prior to performance of baseline breast MRI scan and vacuum assisted biopsy (VAB) of the most dense region of one breast. Ideally 10 cores of breast tissue will be taken. After the onset of the next menstrual cycle subjects will start UA 5mg daily and be reviewed every 4 weeks in trial clinics for blood tests and toxicity assessment. In the 12th week of therapy subjects will have a repeat breast MRI scan followed by either VAB of the contralateral breast or prophylactic mastectomy with harvest of breast tissue (from the predetermined contralateral isodense region from baseline MRI). UA will then be discontinued and one further safety visit planned for 4 weeks post completion (figure 2).

Figure 2: Trial schema

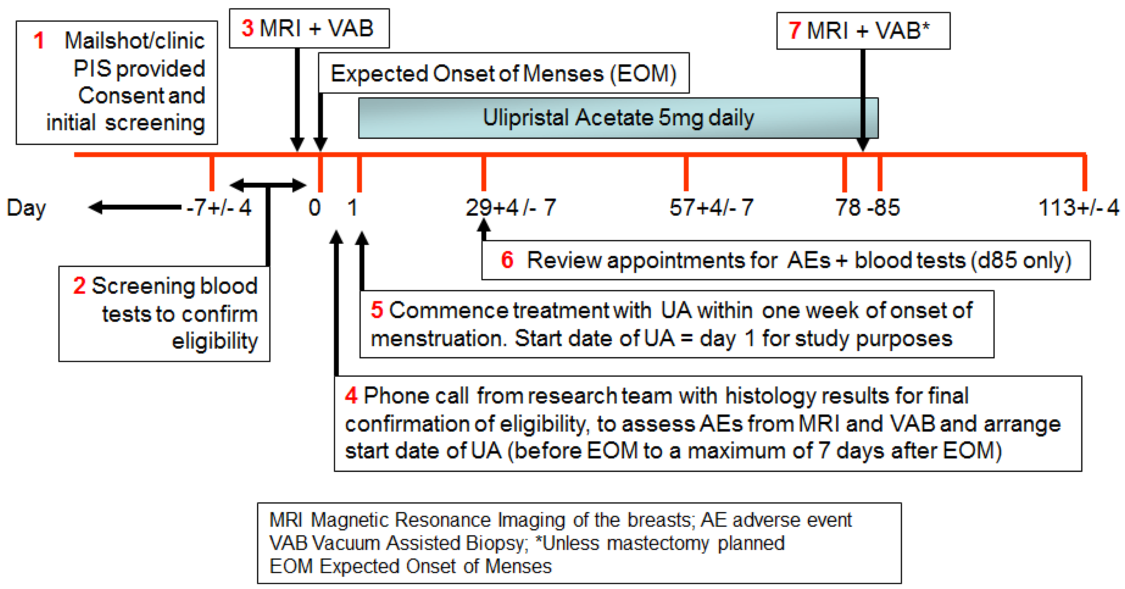

The end of the trial will be when the last participant has completed the 17 week visit – no further data will be collected after this point.

5. Trial medication

5.1 Investigational Medicinal Product

Active treatment: Ulipristal acetate (Esmya) 5mg once a day.

Manufacturer: Gedeon Richter (UK) Ltd.

5.2 Legal status of the drug

This drug is licensed for use in the UK or other countries for the following indications:

- Ulipristal acetate is indicated for pre-operative treatment of moderate to severe symptoms of uterine fibroids in adult women of reproductive age.
- Ulipristal acetate is indicated for intermittent treatment of moderate to severe symptoms of uterine fibroids in adult women of reproductive age.

5.3 Reference safety information and known drug reactions

5.3.1 Reference safety information

The Summary of Product Characteristics (SmPC) is to be used for the reference safety information. The version of this will be the version submitted as part of the MHRA Clinical Trials Authorisation submission package.

Any updates to the SmPC will be reviewed by the Chief Investigator to determine if the new version of the SmPC should be used for the trial – if so a substantial amendment is needed to gain approval from the MHRA for the use of the new SmPC.

The version of the SmPC to be used will be version 14/1/14 – 5/6/15.

5.3.2 Known drug reactions and interaction with other therapies

See Appendix 1: inhibitors/inducers of CYP3A4 not to be taken with UA

5.4 Drug storage, supply and labelling

5.4.1 Dosing Regimen

An oral dose of ulipristal acetate of 5 mg daily was chosen because this drug is only available in oral form and this is an effective dose which induces anovulation in 70-80% of women and a significant fall in serum progesterone. This makes it a suitable test dose for this pilot study.

5.4.2 Drug storage

This trial uses commercially available ulipristal acetate 5mg 28 tablets, which is kept in pharmacy within the clinical trials area at room temperature as per SmPC and standard practice.

5.4.3 Drug supply and accountability

The study medication will be obtained from NHS supplies as per local standard practice and labelled as per annex 13 and specific requirements supplied in the Clinical Trials Application. This will be stored in the clinical trials area.

Pharmacy will dispense the trial medication upon receipt of a suitably signed trial specific prescription and accountability logs will be completed. The original prescription and completed accountability logs will be kept in the pharmacy trials folder. A copy of the prescription will be handed out with the medication to a member of the research team to be filed as appropriate. The participant will be asked to bring all unused medication to the clinic at each visit where it will be checked for compliance and, at the end of treatment, returned to pharmacy.

All unused/expired medication will be only destroyed on site as per local procedures after authorisation by the sponsor.

There won't be post-trial access to IMP once the treatment period finalise.

5.4.4 Preparation and labelling of Investigational Medicinal Product

Labelling of the trial IMP will be performed upon receiving the medication in the pharmacy clinical trials area. This will be done as per local procedures, annex 13 and information submitted and approved by MHRA in the CTA.

5.5 Subject Compliance.

- The study team will record if all the UA has been taken and the reasons why if any tablets have been missed at the day 29, day 57 and day 78 visits. This will be recorded in the CRF.
- Pharmacy will record the amount of medication returned to them.

5.6 Trial restrictions

5.6.1 Acceptable methods of contraception:

- Placement of a non-progestagen releasing intrauterine device
- Barrier methods of contraception: condom or occlusive cap (diaphragm or cervical/vault caps) with spermicidal foam/gel/film/cream/suppository
- Male partner sterilisation (with the appropriate post-vasectomy documentation of the absence of sperm in the ejaculate)
- True abstinence

5.6.2 Non-acceptable methods of contraception:

- progestagen-only pills
- progestagen-releasing intrauterine device
- combined oral contraceptive pills

5.6.3 Pregnancy/genital bleeding

Participants who become pregnant or, develop genital bleeding of unknown aetiology or for reasons other than uterine fibroids on the study must discontinue ulipristal acetate immediately. Note; partial or complete cessation of menstruation is an expected effect of UA therapy. Delayed menstruation and intermittent vaginal bleeding/spotting are expected effects and do not require cessation of therapy.

5.6.4 ALT

If at any point when the ALT is measured it is found to be more than three times the upper limit of normal, UA must be permanently discontinued.

5.7 Concomitant Medication

Certain medication will not be permitted – See Appendix 1: inhibitors/inducers of CYP3A4 not to be taken with UA.

6. **Participant recruitment and eligibility**

Potential recruits will be identified using three approaches:

1. Mailshot of potentially eligible women from the Manchester Family History clinic database.
2. Approach of women attending family history clinics
3. Identification and subsequent approach of women passing through the South Manchester prophylactic mastectomy MDT

6.1. **Inclusion Criteria**

- Premenopausal females aged between 25 and 45 years
- Regular menses defined as date of onset of last menstrual period +/- 3 days of expected
- Willing to use an acceptable method of contraception from screening to 2 weeks after study drug discontinuation (see Restrictions section 5.3 below)
- Known BRCA1 or BRCA2 mutation or moderate to high risk of developing BC defined as >17% lifetime risk from age 20 or >3% risk between 40-50 years
- Ovulatory menstrual cycles defined as serum progesterone ≥15nmol in the luteal phase of the menstrual cycle (2 weeks prior to expected onset of menses).
- Willing and able to provide informed consent to undergo all trial procedures.

6.2. **Exclusion criteria:**

- Personal history of breast, uterine, cervical or ovarian cancer
- Breast feeding within the last 3 months
- Pregnant or planning for pregnancy in the next 6 months. Pregnancy must be excluded with serum βhCG.
- Known hypersensitivity to radiological contrast media
- Known hypersensitivity to ulipristal acetate or any of its excipients (microcrystalline cellulose, mannitol, croscarmellose sodium, talc, magnesium stearate)
- Current treatment with:

o Anti-estrogens (e.g. tamoxifen or raloxifene), GnRH analogue therapy (e.g. goserelin or buserelin) or hormonal contraceptives including androgens such as cyproterone acetate. Such treatments must have been stopped for at least 6 months and regular menstrual cycles resumed.

o Oral corticosteroids at any dose, these must have been stopped for at least 1 month with low likelihood that retreatment will be required

o Antiplatelet or anticoagulant therapy – must have been stopped for at least 7 days and clotting be at satisfactory levels (see below)

o moderate or potent inhibitors of CYP3A4 (for list see appendix)

o potent inducers of CYP3A4 (for full list see appendix 1)

- APTT and PT above the upper limit of the normal institutional ranges. Hb <100g/l and platelet count <$150x10^9$/l. bilirubin, ALT , ALP or LDH >1.5xULN.
- Previous/current diagnosis of hepatitis
- Diagnosis of cirrhosis of the liver

- Co-morbidity that would put the patient at increased risk such as recognised bleeding diathesis, moderate to severe hepatic impairment, moderate or severe renal impairment (eGFR <60 ml/min/1.73m^2), severe asthma not adequately controlled with corticosteroids (note steroid usage precludes trial entry)

- Prior breast enhancement/augmentation surgery

- Genital bleeding of unknown aetiology or for reasons other than uterine fibroids

6.3 Withdrawal of Subjects

Participants have the right to withdraw from the study at any time for any reason. The investigator also has the right to withdraw patients from the study drug in the event of inter-current illness, AEs, SAE's, SUSAR's, protocol violations, administrative reasons or other reasons. It is understood by all concerned that an excessive rate of withdrawals can render the study un-interpretable; therefore, unnecessary withdrawal of patients should be avoided. Should a patient decide to withdraw from the study, all efforts will be made to report the reason for withdrawal as thoroughly as possible. Should a patient withdraw from study drug only, efforts will be made to continue to obtain follow-up data, with the permission of the patient.

The reason for withdrawal will be recorded in the CRF.

If the participant is withdrawn due to an adverse event, the investigator will arrange for follow-up visits or telephone calls until the adverse event has resolved or stabilised.

7. Visit details and methodology

7.1 Visit details

Summarised in the visit schedule in appendix 5.

Screening visit (day – 7 ± 4 days EOM)

During this visit the participant will:

- be consented into the clinical trial
- have demographic and other relevant information documented including date of birth, age at registration, date of onset of last period, average interval between periods (days), estimated date of next period and current method of contraception
- have their medical history documented including BRCA mutation status (BRCA wt, BRCA1 mut, BRCA2 mut) if known and lifetime risk of breast cancer (BC).
- have their weight and height recorded
- have any baseline symptoms recorded
- have their current concomitant medications recorded.
- have blood samples taken for serum progesterone, APTT/PT/FBC of which Hb and platelets will be recorded in the CRF /U+E of which serum creatinine will be reviewed to determine eGFR levels/LFT of which bilirubin, ALT, AST, ALP, LDH will be recorded in the CRF/ Lipid profile of which total cholesterol will be recorded in the CRF and TFT of which TSH and T4 will be recorded in the CRF . A Serum βhCG pregnancy test will also be perfomed. Serum and plasma samples will also be stored for investigational assays.
- have their samples prepared and stored as described in sections 8 and 9.
- have their eligibility for MRI scans assessed (contraindications to MRI, such as intracranial aneurysm clips, implanted electrical devices and intra-ocular metallic foreign bodies will exclude participants from undergoing an MRI but they can continue with all other trial procedures. In addition, should a participant not fulfil the size/weight requirements of the scanner or if the scanner breaks down resulting in a participant not having an MRI scan they can continue with other trial procedures).
- The importance of using contraception throughout the trial will be emphasised.
- Eligibility will be determined.

MRI visit (day – 7 ± 4 days EOM to OM)

During this visit:

- The participant will be asked if they are happy to continue in the trial
- The participant will undergo an MRI scan

If a participant is unable to tolerate the MRI scan, for example due to claustrophobia, she may continue on study and undergo all other trial procedures. In addition, should a participant not fulfil the size/weight requirements of the scanner or if the scanner breaks down resulting in a participant not having an MRI scan they can continue with other trial procedures.

VAB visit (day – 7 ± 4 days EOM to OM)

During this visit:

- Eligibility must have been confirmed by the PI or delegated individual prior to the VAB and provision of the IMP to the participant.
- The participant will be asked if they are happy to continue in the trial
- Any changes to the participant's concomitant medications will be documented
- Adverse events from the MRI visit or any other adverse events will be documented
- 3x 28 day pack supply of 5mg UA will be supplied to the participant who will be instructed to wait for a telephone call from the study team before starting the medication. Participant will also be given a study card with the medication supply
- The patient will undergo breast electrical impedance measurement
- The patient will undergo the VAB of one breast
- H&E pathological analysis of breast tissue to exclude invasive cancer and Ductal carcinoma in situ (DCIS), If the results of the H&E pathological analysis are not clear, one of the Study Doctors will contact the participant to discuss this with them.

If invasive cancer or DCIS are identified the participant will be discussed at the UHSM NHS breast Multi-disciplinary Meeting within 1 week and managed according to standard NHS practice thereafter. Such participants will not be eligible to continue on study.

Telephone call to patient regarding histology results and onset of menses

- The participant will be asked if they are happy to continue in the trial and the importance of using contraception throughout the trial will be emphasised.
- Adverse events from the VAB visit or any other adverse events will be documented
- The participant will be asked if they have started their period

- If the participant has started their period, and there is no evidence of cancer or DCIS on the breast biopsy, they will be instructed when to start study medication . This will be within 1 week of onset of menstruation (OM), in line with the UA SPC.
- If the participant has not started their period then they will be instructed to contact the research team at the OM or on the next working day (M-F) and they will be advised when to commence study medication (UA). If the participant has not phoned by a maximum of 5 days past her EOM then the research team will contact her to ask if she has started her period. If so then she will be asked the date of OM and will be advised when to start UA. If the participant has still not started their period she will be asked to contact the research team again as above. In line with the UA SPC treatment should be started within 1 week of OM.
- The day the study medication is started will be counted as day 1 for visit schedule purposes
- The first visit on treatment (visit 1) will be scheduled during this phone call to fall on day 29 +4/-7 days
- If the results of the H&E pathological analysis were not clear, one of the Study Doctors will contact the patient to discuss this with them

Visit 1 Day 29 + 4 / - 7 days (week 5)

During this visit the participant will:

- be asked if they are happy to continue in the trial and the importance of using contraception throughout the trial will be emphasised.
- have changes in concomitant medications documented
- have a urine pregnancy test performed
- have blood samples taken for LFT of which bilirubin, ALT, ALP, LDH will be measured. If ALT is more than three times the upper limit of normal, UA must be permanently discontinued.
- have their weight recorded
- have any adverse events documented and be asked whether they have had any vaginal bleeding.
- have a drug accountability check performed

Visit 2 Day 57 + 4 / - 7 days (week 9)

During this visit the participant will:

- be asked if they are happy to continue in the trial and the importance of using contraception throughout the trial will be emphasised.

- have any changes to concomitant medications documented
- have a urine pregnancy test performed
- have their weight recorded
- have blood samples taken for LFT of which bilirubin, ALT, ALP and LDH will be recorded. If ALT is more than three times the upper limit of normal, UA must be permanently discontinued.
- have any adverse events documented and be asked whether they have had any vaginal bleeding.
- have a drug accountability check performed

Visit 3 Day 78-85 (week 12)

During this visit the participants will:

- be asked if they are happy to continue in the trial. and the importance of using contraception throughout the trial will be emphasised.
- have blood samples taken for serum progesterone, APTT/PT/FBC/U+E/LFTs of which bilirubin, ALT, ALP and LDH will be recorded /LDH, lipid profile and TFT. If ALT is more than three times the upper limit of normal, UA must be permanently discontinued Research samples will also be taken for storage.
- have their samples prepared and stored as described in sections 8 and 9
- have any changes to their concomitant medications documented
- have a urine pregnancy test performed
- have their weight recorded
- have any adverse events documented and be asked whether they have had any vaginal bleeding.
- have a drug accountability check performed

MRI 2 visit Day 78-85

During this visit the participant will :

- be asked if they are happy to continue in the trial
- undergo an MRI scan

VAB 2 visit/ Prophylactic Mastectomy visit Day 78-85

During this visit the participant will:

- be asked if they are happy to continue in the trial
- have any changes to their concomitant medications documented
- the patient will undergo breast electrical impedance measurement
- have any adverse events documented including those from MRI 2 and be asked whether they have had any vaginal bleeding.
- undergo the VAB of the breast that was not biopsied at baseline or Prophylactic Mastectomy

Telephone call after VAB 2 visit Day 78-85

- The participant will be asked if they are happy to continue in the trial
- Adverse events from the VAB visit or any other adverse events will be documented

Visit 4 Day 113 ± 4 days (week 17)

During this visit the participant will:

- be asked if they are happy to continue in the trial
- have adverse events from the VAB visit and any other adverse events documented
- have any changes in their concomitant medications documented
- have their weight recorded
- have blood samples taken for LFT of which bilirubin, ALT, ALP, LDH will be measured
- be asked if they have started their menstrual period

Should a participant experience treatment emergent clinical symptoms or signs of liver disease whilst participating in the study they will be advised to contact the research team as soon as possible for assessment which will include repeat LFTs of which bilirubin, ALT, ALP and LDH will be recorded. Therefore, assessments of this nature could be conducted outside of the scheduled study visits.

Retrospective collection of weight and height

Participants who have completed the study or did not have their height recorded at their baseline appointment will be contacted by a member of the team and asked for their height, this will then be recorded in the participant's notes.

Where weight was not collected, the weight used to calculate the dose of contrast at MRI will be used.

8 Methodology

8.1 Timing of study entry by luteal phase progesterone

Luteal progesterone levels will be checked 7 days prior to the expected onset of the next menstrual cycle. The serum progesterone level must be ≥15nmol to confirm an ovulatory menstrual cycle for further trial related procedures to take place. If the level is <15nmol then one further measurement may be taken in the following menstrual cycle (again at day 7 prior to expected onset of menses). If the progesterone level on the second occasion is <15nmol then screening has failed and the woman will not be permitted to enter the study.

8.2 Biopsy and sample processing protocol

8.2.1 Vacuum assisted biopsy (VAB)

10G vacuum assisted biopsy (VAB) under local anaesthetic will be employed to obtain sufficient tissue for all planned analyses. A single 5mm skin incision will be made and a planned total of 10 tissue cores taken under ultrasound guidance. The following procedure will be adopted with each core and the samples transported to the Breast Biology laboratory within 4 hours from harvest:

- Cores 1 and 2 will be snap frozen separately in liquid nitrogen for subsequent nucleic acid extraction.
- Core 3 will be placed in a 10% formalin for subsequent paraffin embedding and IHC analyses. A section will be taken for H&E staining for the pathological analysis of breast tissue to exclude invasive cancer and DCIS by the UHSM pathology department.
- Core 4 will be placed in a separate pot of 10% formalin for subsequent paraffin embedding for stromal/collagen analyses
- Cores 5-10 (or up to 10 as can be retrieved) will be placed into DMEM medium at 4^0C for subsequent clonogenic and FACS assays.

Published data indicate an average core weight of ~100mg (Berg 1997; Burbank 1997) and our own data demonstrate mean cell counts of ~300,000 per 500mg of tissue, indicating that with 5-7 cores there will be sufficient cells for clonogenic and FACS analysis (Clarke 2005; Eirew 2010).

8.2.2 Prophylactic mastectomy samples

For women planning to undergo prophylactic mastectomy, no second VAB biopsy is required. However, these women will require take UA up to and including the day of their operation. At mastectomy an orientation clip will be placed as per normal practice. Following the mastectomy procedure the excised breast will be transported fresh to the breast pathologist at UHSM and the area best correlating to the most dense area on mammography (broadly identified by breast quadrant) will be excised and divided into liquid nitrogen, formalin and culture medium as described in 4.5.1 above. At least 1gm of

tissue will be harvested for analysis although there is no restriction on the amount of tissue that can be returned to the Breast Biology Group laboratory in these cases.

8.3 Radiological Assessments

Baseline mammography will be analysed using Volpara™ and the breast density will be correlated with the subsequent MRI-derived measurements. Serial MRI will be used to correlate biological with radiological changes. The fibroglandular distribution for each breast at baseline MRI will enable identification of areas of comparable density for biopsy at each time point. The MRI examinations will be performed in the 7 days prior to the expected onset of menses on a 1.5T Philips MRI scanner using a dedicated multichannel breast coil. A dynamic contrast-enhanced protocol will be used with the recommended body weight-adjusted dose (0.1mmol/kg) of intravenous gadolinium-containing contrast medium delivered via a pump injector. The images will be viewed on a modality workstation and/or PACS workstation. Further image analysis will be performed on a workstations running image analysis software. Image segmentation will be performed to determine fibroglandular and overall breast volumes. Background parenchymal enhancement will be measured both visually and using standardised regions of interest (ROIs). In addition, Ktrans, ve (extracellular/leakage space), vp (vascular volume), fibroglandular apparent diffusion coefficient and fractional anisotropy, water/fat ratio and T1 and T2 relaxation times will be measured.

Ultrasound elastography will also be performed immediately prior to the vacuum biopsies. This will be with a Siemens S3000 ultrasound machine utilising acoustic radiation force imaging (ARFI) with a 9Mz probe. Measurements of fibroglandular and fat tissue stiffness will be performed in each of the four quadrants of both breasts, targeting the densest and least dense area in each quadrant respectively. Changes in tissue stiffness between pre- and post-treatment scans will be explored and correlated with the MRI data.

Another potentially useful measure of responsiveness of the breast to preventive therapy is change in the electrical conductance of the breast. It depends on differences in electrical impedance between fat and glandular/stromal tissue. It has not been widely investigated but has the potential value of simplicity and repeatability and, depending on further studies, has the potential to detect change early.

8.4 Endpoints

8.4.1 Primary endpoint

The change in the proliferation of normal breast epithelium with UA treatment, assessed as the percentage of epithelial cells staining positively for nuclear Ki67 before and on treatment.

8.4.2 Secondary endpoints

Secondary endpoints are the change in the relevant outcome with UA therapy across the whole population of participants:

- Percentage of luminal, basal and mixed colonies by morphological analysis of adherent feeder layer assay
- Percentage of luminal progenitor cells (EPCAM$^+$/CD49f$^+$) by FACS analysis
- Tissue stiffness assessed as the reduced indentation modulus by atomic force microscopy
- Mean tissue section percentage fibrillar collagen assessed by picrosirius red staining and polarised light microscopy
- Background parenchymal enhancement assessed by magnetic resonance imaging (MRI)
- The side effect profile of UA in this patient population assessed by CTCAE v4.03
- The relative change in Ki67 with UA treatment between those with and without known mutation in BRCA1/2 genes

8.5 Exploratory endpoints

The changes in expression of individual genes and key pathways induced by UA therapy

The changes in key stem cell and PgR target proteins induced by UA therapy

The changes in fibroglandular volume and other MRI/US elastography biomarkers

The changes in fibroglandular and fat tissue stiffness assessed by ultrasound elastography and acoustic radiation force imaging (ARFI)

The changes in breast impedance measured at baseline and 3 months.

9 Specific protocols for endpoint analyses

9.1 Ki67 analysis

Samples will be transported to the Breast Biology Group laboratories in 10% formalin. After 24+/-6 hours in formalin (and with the exact time documented) samples will be embedded in paraffin in the pathology core facility at the CRUK Manchester Institute (CRUK-MI). Samples will be embedded in optimal cutting temperature cryo-sectioning media and snap frozen into a 2.5cm mould with liquid nitrogen cooled isopentane. Frozen sections (5um) will be cut at -20°C, air dried, washed in distilled H_2O and stored at 4°C prior to use.

Paraffin sections will undergo antigen retrieval at 115℃ for 3 min (microwave processor) in Tris–EDTA buffer (pH 9.0) followed by staining with anti-MIB1 antibody at 1:200 dilution for 1 hour incubation at 37 ℃. Following appropriate and uniform secondary antibody application and signal amplification the slides will be manually scored with the operator blinded to the sample time point (baseline or on treatment). For final analysis all samples will be stained and analysed at the same time to avoid between batch variability.

However, local optimisation of the techniques using some of the samples may occur prior to this. The operator will count at least 1000 cells per sample where possible and score the percentage with nuclear Ki67 staining.

9.2 Clonogenic assays

Clonogenicity will be assessed through adherent feeder layer and suspension mammosphere assays in unsorted cells. For adherent clonogenic assays mammary cells (2,000-5,000 cells) from individual patients will be plated with irradiated fibroblasts (a generous gift from our collaborator J Stingl, Cambridge) and cultured in SF7 medium containing 5% FBS + 20uM ROCK inhibitor at 5% oxygen conditions for 7-10 days. Colonies will be fixed and typed (basal/luminal/mixed) by morphological analysis as previously described (Clarke 2005, Eirew 2010). FACS analysis will be performed on freshly isolated single cell suspensions (~200-300,000 cells) by first excluding lineage+ cells (CD45 – haematopoietic, Ter119 –erythrocytes, CD31 – endothelial), and then identifying the proportion of basal cells (CD49f+/EPCAM-/lo), luminal progenitor cells (CD49f+/EPCAM+) and differentiated luminal cells (CD49f-/EPCAM+) before and after therapy with UA.

9.3 Morphological analysis, tissue stiffness and collagen analyses

Samples will be transported to the Breast Biology Group laboratories in 10% formalin. After 24+/-6 hours in formalin (and with the exact time documented) samples will be embedded in paraffin in the pathology core facility at the CRUK Manchester Institute (CRUK-MI). Samples for morphological analysis will be transferred to the UHSM pathology laboratories. Samples for stromal/collagen analyses will be transported to the laboratory of Dr Michael Sherratt in the Faculty of Life Sciences, The University of Manchester.

H&E sections will be assessed by an accredited pathologist for evidence of invasive cancer or DCIS. Any changes will be investigated further using additional samples and more conclusive testing as per standard routine practice.

Tissue stiffness

Micro-indentation of peri-ductal breast tissue will be carried out using a Bioscope Catalyst AFM (Bruker, Coventry, UK) mounted onto an Eclipse T1 inverted optical microscope (Nikon, Kingston, UK) fitted with a spherically tipped cantilever (nominal radius and spring constant of 1µm and 3N m^{-1} respectively: Windsor Scientific Ltd., Slough, UK,) running Nanoscope Software v 8.15 (Bruker, Coventry, UK). The local reduced modulus will be determined for each of 400 points in a 25x25µm region, indented at a frequency of 1 Hz with lateral spacing of 1.25 µm. The extend curve will be used in conjunction with a contact point based model to calculate the reduced modulus for each indentation. For each biological sample, three 25μm^2 regions and hence 1200 force curves will be collected. A baseline correction will be applied to each curve before a force fit is applied using trh Herzian (spherical) model and a maximum force fit of 70%. Once all 400 force curves have been generated, a quality control will be applied whereby any force values falling more than two standard deviations away from the mean value will be discarded in order to account for failed indents (Crick 2007; Graham 2010).

Collagen analysis

FFPE samples (see above) will be stained with picrosirius red (PSR) for a 1 hour incubation with 0.1% sirius red F3BA in saturated aqueous picric acid at pH 2 followed by clearing in 0.1% acetic acid, dehydration and mounting in DPX (Graham 2011). When visualised under cross-polarised light, the resultant collagen-associated birefringence will be assessed semi-quantitatively against total tissue area.

9.4 Magnetic Resonance Imaging

Previous studies have demonstrated rapid reductions in background parenchymal enhancement (BPE) with SERM therapy in premenopausal women and greater reduction in BPE than FGV with transition from pre- to post-menopausal. We hypothesise that BPE will also reduce in women sensitive to the effects of the antiprogestin UA with three months of treatment. The primary imaging analysis will, therefore, be the change in BPE associated with UA therapy within individuals, scored according to the BI-RADS categories (minimal, mild, moderate and marked).

9.5 Exploratory analyses

Gene expression

To determine gene expression using unbiased methods before and after treatment, fresh frozen tissue from patients will be subject to RNA extraction and Poly(A) RNA-Sequencing and analysed using appropriate BioConductor packages to identify pathways and key nodes perturbed by UA. Validation of expression changes in selected individual genes will be performed using qRT-PCR and IHC. Further fresh frozen tissue will be stored for future DNA analysis.

Immunohistochemistry and tissue microarray (TMA)

Cores will also be taken from the FFPE samples to construct a tissue microarray (TMA). Staining of the TMA will be performed in accordance with validated protocols for known regulators of PgR action (ER, PRA/B ratio, RANKL and Wnt4) and stem cell function (ALDH1a1 and 1a3, Notch-1 and Notch 4-ICD, Hes1 and Hey2, Lef1, Axin2, Lgr4/5 and nuclear beta catenin). These parameters will be analysed in both the epithelial and stromal cell populations in tissue sections. Baseline and dynamic change in subnuclear localisation of PR with UA will be examined as a biomarker of antiprogestin activity (Scarpin 2009). To investigate the effects of UA on matrix composition expression of collagen, proteoglycans, focal adhesion complex signals (eg paxilin and phospho-FAK) will be assessed.

Imaging

MRI has the advantage that, unlike mammography, it can be repeated early in the course of treatment as it does not use ionising radiation. It is crucial that the biological correlates of these imaging data are examined in detail in this and future prevention studies. We will seek to develop an objective standardised method for assessment of BPE to be taken forward to future large scale assessments of risk in the national high risk screening programme. This will include correlation of visually assessed and ROI-based measures of BPE with the other factors. We also propose to measure the apparent diffusion coefficient (ADC) which reflects the freedom of movement of water molecules within the tissue and is related to tissue cellularity and water content. There is a paucity of data on changes in ADC in response to hormonal therapy, but ADC values are lower in post-menopausal women (O'Flynn 2012) and one study has reported small changes during the menstrual cycle (Partridge SC 2001). Diffusion tensor imaging will give ADC directional information (fractional anisotropy).

Ultrasound elastography will also be performed immediately prior to the vacuum biopsies. This will be with a Siemens S3000 ultrasound machine utilising acoustic radiation force imaging (ARFI) with a 9Mz probe. Measurements of fibroglandular and fat tissue stiffness will be performed in each of the four quadrants of both breasts, targeting the densest and least dense area in each quadrant respectively. Changes in tissue stiffness between pre- and post-treatment scans will be explored and correlated with the MRI data.

Blood

Both serum and plasma samples will be taken and stored for subsequent analysis at baseline and 12 week time points ie when bloods are already being taken, to prevent additional venepunctures. The precise analyses to be performed are not know at this time, however, the storage of both serum and plasma will facilitate detailed hormonal and proteomic analyses. 10ml serum samples will be prepared in standard tubes. Plasma samples will be collected in EDTA tubes and spun at 2000rpm for 10 minutes at room temperature before storage at -80^{0}C in the on-site Nightingale Centre -80^{0}C freezer (UHSM).

10 Statistical considerations

In a previous study using mifepristone and FNA of the normal breast (Engman 2008) a significant reduction in Ki67 was seen with only 8 patients in the treatment group (5.76±1.42 vs 0.79±0.27 p=0.012). In contrast no difference was seen in 6 placebo treated women. A 30 subject study will thus provide sufficient subjects to explore variability in response both by primary endpoint (Ki67) but also secondary clonogenic, stromal and radiological endpoints. We will seek to recruit approximately half of the group with and half without BRCA mutations given the potential differential sensitivity in luminal progenitors described above.

Differences between baseline and on treatment Ki67 (ΔKi67) across the whole population (primary endpoint analysis) will be assessed using the Wilcoxon signed rank test. Differences in ΔKi67 between women with and without known BRCA mutations and other

exploratory analyses (such as development of amenorrhoea vs not and sample dichotomised by median age and on treatment progesterone levels) will be assessed using the Mann–Whitney U-test.

Secondary endpoint analyses will be conducted in a similar manner using the relative expression levels of individual genes by qRT-PCR (ΔCT value) or proteins by immunohistochemistry using percentage cells staining positively before and after therapy (for individual proteins this may be in the nucleus, cytoplasm, cell membrane or combination of these as appropriate); semiquantative analysis of fibrillar collagen by picrosirius red staining and the proportion of luminal progenitors in clonogenic and FACS analyses (CD49f+/EPCAM+). The categorical Bi-RADs values for MRI scans will be analysed using appropriate non-parametric tests.

As one of the primary aims of the study is to identify imaging biomarkers of anti-progestin activity we will classify MRI changes according to reduction in background parenchymal enhancement (BPE) with UA treatment. Serial MRI studies demonstrate moderate to marked reduction in BPE in 50% of women transiting menopause and a similar proportion with increased BPE in the luteal vs follicular phase of the menstrual cycle. As BPE is associated with BC risk we hypothesise that any reductions in BPE with UA will correlate with reduced BC risk. We will correlate UA induced changes in BPE (and also FGV and ADC) with changes in clonogenicity and proportion of cells in each compartment (basal, luminal progenitor and luminal). Similarly, any changes in stromal composition, organisation or stiffness assessed by AFM will be correlated with changes in MRI parameters.

11 Trial Risk Category, Potential benefits and risks for participating patients.

This trial is categorised (based on MRC/DH/MHRA joint project 10th October: Risk-adapted Approaches to the Management of Clinical Trials of Investigational Medicinal Products) as:

- Type B = Somewhat higher than the risk of standard medical care

Justification: Ulipristal acetate (UA) 5mg is an established licensed drug in the UK for use in the treatment of uterine fibroids. UA will be used in a new indication in this trial for breast cancer prevention and has therefore been categorised as type B.

11.1 Benefits:

It is unlikely that any physical benefits will result from participation in this trial. Even if the hypothesis that antiprogestin therapy may reduce BC risk is correct, any risk reduction with only 3 months of therapy is likely to be negligible.

11.2 Risks:

The gadolinium contrast agent given during the MRI scans can cause mild nausea and rarely headache and/or vomiting. Allergic reactions are rare (~1 in 10,000) and generally respond well to standard treatment. All contrast agent injections will be supervised by a medical practitioner.

By participating in this study women will have at least one and some will have 2 vacuum assisted biopsies of the breast(s). The risks associated with VABs including pain, bruising, haematoma, bleeding, infection and scarring of the breast are detailed in the patient information sheet. To mitigate against these risks only experienced radiologists will perform study VABs (AM and YL). Additional physical harms come from the blood tests that would otherwise not be performed.

Should pregnancy occur in any of the trial participants, the risk to the foetus is unknown. However UA is known to induce anovulation and therefore pregnancy is less likely than without the use of the drug.

Risks mitigations:
The MRI unit performing the scans have medical cover and medication available on site should a contrast reaction occur. Participants who have an eGFR <60 ml/min/1.73m2 will be excluded. A serum pregnancy test will be carried out at screening. Urine pregnancy tests will then be carried out at each 4 week visit through the trial and the importance of using contraception will be emphasised at multiple points throughout the trial. To prevent recruitment of women with breast cancer a sample of the baseline VAB biopsy will be examined by an accredited breast pathologist before trial eligibility is confirmed.

12 Forms and procedures for collecting data

12.1 Case report forms

Data will be abstracted from the family history medical records onto case report forms and entered into a trial specific database. Access to the ongoing trial data will be restricted to the trial team and R&D.

Data will be transcribed into an Excel spreadsheet and data verification will be performed including a quality control check of 10% of the data added into the statistical and analytical software prior to analysis as described in appendix 2.

Access to the final dataset will be restricted to the trial team and R&D. Source data for the trial will be: defined in the source data log. Source data verification will be performed as part of the monitoring for the trial and will be described in the monitoring plan.

All patients require the CRF to be completed within 1 month of entry into the study. Research staff must ensure that all source data and trial related documentation are

accurate, complete, maintained and accessible for monitoring and audit purposes.

Original consent forms must be dated and signed by both patient and investigator and will be kept together in a central log together with a copy of the specific patient information sheet(s) given at the time of consent

Copies of CRFs will be retained for 15 years to comply with international regulations

12.2 Data flow

Records of numbers of patients approached, assessed for eligibility, registered and lost to follow-up will be recorded as per CONSORT guidelines.

CRFs will be developed with the CI, trial management team, statistician and data manager to ensure that the data management system supports both the research aims of the study and regulatory requirements. Any CRF input amendments must be signed and dated by study staff. The data management system will be fully validated, including the provision of test data and supporting documentation. The system will be backed-up offsite every 24 hours.

Each participant will be assigned a trial identity code number, allocated for use on CRFs, other trial documents and the electronic database. The documents and database will use centre initials date of birth (dd/mm/yy) and patient number.

CRFs will be treated as confidential documents and held securely in accordance with regulations. The investigator will make a separate confidential record of the participant's name, date of birth, local hospital number or NHS number, and Participant Trial Number (the Trial Recruitment Log), to permit identification of all participants enrolled in the trial in accordance with regulatory requirements and for follow-up as required.

Completion of CRFs shall be restricted to those personnel approved by the Chief or local Principal Investigator and recorded on the 'Trial Delegation Log'.

All paper forms shall be filled in using black or blue ballpoint pen. Errors shall be crossed out (but not obliterated by using correction fluid) and the correction inserted, initialled and dated. Corrections should be made legibly and initialled and dated by approved personnel. The reasons for significant changes must be provided.

If any data are not available, omissions should be indicated on the case report forms. The NHS Code of Confidentiality will be followed for this study.

Sensitive data stored on NHS or University computers will be encrypted to applicable NHS standards (AES256). The results of the study may be published in the medical literature but patient identity will not be revealed.

The trial data and documentation will be archived as per the UHSM SOP11 Archiving.

12.3 What to record in the participant family history medical records

This is described in UHSM SOP4 Study conduct.

13 Safety reporting and pharmacovigilance

The Principal Investigator is responsible for ensuring that all site staff involved in this trial are familiar with the content of this section and UHSM SOP2a Safety Reporting for CTIMPs conducted at UHSM.

The following definitions are in accordance with the Medicines for Human Use (Clinical Trials) Regulations 2004 (SI2004/1031)(as amended) and EU Directive 2001/20/EC.

TERM	DEFINITION
**Adverse Event**<br><br>**(AE)**	Any **untoward medical occurrence** in a subject to whom a medicinal product has been administered, including occurrences which are not necessarily caused by or related to that product.
**Adverse Reaction (AR)**	Any **untoward and unintended response in a subject to an investigational medicinal product** which is related to any dose administered to that subject
**Serious adverse event (SAE), serious adverse reaction (SAR) or unexpected serious adverse reaction**	Any **adverse event or adverse reaction** that:<br><br>- **Results in death**<br>- **Is life-threatening***<br>- **Requires hospitalisation** or prolongation of existing hospitalisation**<br>- **Results in persistent or significant disability or incapacity**<br>- **Consists of a congenital anomaly or birth defect.**<br><br>Important medical events*** may also be considered serious if they jeopardise the subject or require an intervention to prevent one of the above consequences.<br><br>The term life-threatening in the definition of serious refers to an event in which the patient was at risk of death at the time of the event; it does not refer to an event which hypothetically might have caused death if it were more severe.

**Suspected unexpected serious adverse reaction (SUSAR)**	A serious adverse reaction, the nature and severity of which is **not consistent with the information about the medicinal product** in question set out:<br>- In the case or a product with a marketing authorisation, in the summary of product characteristics for that product.<br>- In the case of any other investigational medicinal product, in the investigator's brochure relating to the trial in question.
**Reference safety information**	The information used for assessing whether an adverse reaction is expected. This is contained in either the **investigator's brochure** or the **summary of product characteristics**.

***Note:** The term 'life-threatening' in the definition of serious refers to an event in which the trial participant was at risk of death at the time of the event or it is suspected that use or continued use of the product would result in the subjects death; it does not refer to an event which hypothetically might have caused death if it were more severe.

**** Note:** Hospitalisation is defined as an inpatient admission, regardless of the length of stay, even if the hospitalisation is a precautionary measure for continued observation. Pre-planned hospitalisation e.g. for pre-existing conditions which have not worsened, or elective procedures, does not constitute an SAE.

***** Note:** other events that may not result in death, are not life-threatening, or do not require hospitalisation, may be considered as an SAE when, based upon appropriate medical judgement, the event may jeopardise the participant and may require medical or surgical intervention to prevent one of the outcomes listed above.

Definition of Adverse Events for the trial

In all cases AEs and / or laboratory abnormalities that are critical to the safety evaluation of the participant must be reported to the Sponsor; these may be volunteered by the participant, discovered by the investigator questioning or detected through physical examination, laboratory test or other investigation.

All SAEs/SARs/SUSARS must be reported to the Sponsor

Expected Serious Adverse Events for the trial

Any expected SAEs for the disease and trial drug / intervention?

Adverse events that do not require reporting

Normal menstrual bleeding, including vaginal spotting, will not be classed as an adverse event and therefore does not need reporting. It will be recorded in the participants' family history notes as part of the study visit information.

Causality Assessments

The process for causality assessments is described in **UHSM SOP 2a Safety Reporting for CTIMPs conducted at UHSM.**

Expectedness Assessments

The Chief Investigator on behalf of the Sponsor will assess each SAE to perform the assessment of expectedness.

The process for expectedness assessments is described in **UHSM SOP 2a Safety Reporting for CTIMPs conducted at UHSM.**

The expectedness assessment should be made with reference to the current Reference Safety Information (RSI) for the IMP in this trial (Ulipristal Acetate SPC). Expectedness decisions must be based purely on the content of the RSI; other factors such as the participant population and participant history should not be taken into account. Expectedness is not related to what is an anticipated event within a particular disease.

SAEs which add significant information on specificity or severity of a known, already documented adverse event constitute unexpected events. For example, an event more specific or more severe than that described in the RSI is considered unexpected.

13.1 Recording and reporting of SAEs AND SUSARs

UHSM SOP 2a Safety Reporting for CTIMPs conducted at UHSM must be used for safety reporting.

AEs, ARs, SAEs,SARs and SUSARs must be recorded and reported as follows:

- For AEs / SAEs – from consent until the time of the last participant visit
- For ARs / SARs and SUSARs – from the 1st IMP dose until the time of the last participant visit

All AEs must be recorded in the participant's CRF in the AE log and in the patient's family history notes. SAEs/SARs/SUSARs must be recorded in the participant's CRF in the AE log and in the patient's family history medical records and must be reported to the Sponsor and other relevant departments/individuals **within 24 hours** of the research staff becoming aware of the event according to the instructions in **SOP 2a Safety Reporting for CTIMPs conducted at UHSM must be used for safety reporting**.

(S)AEs and (S)ARs will be evaluated for duration and intensity according to National Cancer Institute Common Terminology Criteria for Adverse Events V4.0 (NCI-CTCAE) or MedDRA (Medical Dictionary for Regulatory Activities)

Investigator reports of suspected SARs willl also be reported by the CI or their delegate to Gedeon Richter (manufacturer).

13.2 Responsibilities for safety reporting

Principal Investigator (PI), Co-investigators, Study doctors on the delegation log:

- Checking for AEs and ARs when participants attend for treatment / follow-up.
- Using medical judgement in assigning seriousness, causality and expectedness using the Reference Safety Information approved for the trial. The Principal Investigator, Co-investigators or Study doctor designated to carry out this function on the trial delegation log will assess each SAE to determine the causal relationship with the IMP, and will answer 'yes' or 'no' to the question "Do you consider that there is a reasonable possibility that the SAE may have been caused by the IMP?" A guide to the interpretation of the causality question is found in Appendix 1 of this clinical trial protocol.
- Ensuring that all SAEs and SARs (including SUSARs) are recorded and reported to the Sponsor within 24 hours of becoming aware of the event and provide further follow-up information as soon as available.
- Ensuring that AEs and ARs are recorded and reported to the Sponsor in line with the requirements of the protocol and *2a Safety Reporting for CTIMPs conducted at UHSM*.

Chief Investigator (CI) / delegate or independent clinical reviewer:

- Clinical oversight of the safety of patients participating in the trial, including an ongoing review of the risk / benefit.
- Using medical judgement in assigning seriousness, causality and expectedness of SAEs where it has not been possible to obtain local medical assessment. The Chief Investigator, Principal Investigator, Co-investigators or Study doctor designated to carry out this function on the trial delegation log will assess each SAE to determine the causal relationship with the IMP, and will answer 'yes' or 'no' to the question "Do you consider that there is a reasonable possibility that the SAE may have been caused by the IMP?" A guide to the interpretation of the causality question is found in Appendix 1 of this clinical trial protocol.

- Using medical judgement in assigning expectedness. The Chief Investigator, on behalf of the Sponsor, will assess each SAE to perform the assessment of expectedness. ***If the CI is unavailable the Sponsor will find an alternative medically qualified individual with the appropriate knowledge to perform this task***.
- Immediate review of all SUSARs.
- Review of safety signal analysis and SAE trends in accordance with the SOP 2c Safety Monitoring for UHSM sponsored CTIMPS and the Risk Management Plan for UHSM Sponsored CTIMPs.
- Preparing the clinical sections and final sign off of the Development Safety Update Report (DSUR).
- Expedited reporting of SUSARs to the Competent Authority (MHRA in UK) and REC within required timelines (delegated by the Sponsor– *this function can be given to the Clinical Trials Manager/other suitable individual but the CI remains accountable to the Sponsor)*.
- Checking for (annually) and notifying PIs of updates to the Reference Safety Information for the trial (delegated by the Sponsor – *this function can be given to the Clinical Trials Manager/other suitable individual but the CI remains accountable to the Sponsor)*.
- Reporting safety information to the Sponsor oversight committees identified for the trial (Data Monitoring Committee (DMC) and / or Trial Steering Committee (TSC)) according to the Risk Management Plan for UHSM Sponsored CTIMPs – *this function can be given to the Clinical Trials Manager/other suitable individual but the CI remains accountable to the Sponsor)*.
- The CI must review all the safety data for the trial and complete the relevant sections of the Safety Monitoring report which is submitted to Sponsor Oversight Committee on a monthly basis.
- Inform Gedeon Richter (manufacturer) of any SUSARs.

Sponsor:

- Central data collection and verification of all reported SAEs, SARs and SUSARs onto a database.

Trial Management Group (TMG):

See section 16.1 below.

13.3 Out of hours contact arrangements

This will be set up according to the relevant UHSM SOP: SOP 27 Out of Hours.

13.4 The type and duration of the follow-up of subjects after adverse events.

Adverse events will be followed up until the safety visit 4 weeks after the last dose of IMP.

13.5 Reporting urgent safety measures

UHSM SOP 2d Urgent Safety Measures must be followed.

If any urgent safety measures are taken the CI/Sponsor shall immediately and in any event no later than 3 days from the date the measures are taken, give written notice to the MHRA and the relevant REC of the measures taken and the circumstances giving rise to those measures.

13.6 Notification of deaths

Deaths that are assessed to be caused by the IMP will be reported to the sponsor. This report will be immediate.

13.7 Pregnancy reporting

SOP2a Safety Reporting for CTIMPs conducted at UHSMs must be followed.

- All pregnancies within the trial (either the trial participant or the participant's partner) should be reported to the Chief Investigator and the Sponsor using the relevant Pregnancy Reporting Form within **within 24 hours of learning of the event**.
- Pregnancy is not considered an AE unless a negative or consequential outcome is recorded for the mother or child/foetus. If the outcome meets the serious criteria, this would be considered an SAE.

Follow-up of pregnant subject: Any pregnancy will be monitored as deemed appropriate depending on the informed consent given.

Follow-up of child born to a pregnant trial subject, or to the partner of a male trial subject: Any pregnancy will be monitored as deemed appropriate depending on the informed consent given.

13.8 Overdose

- Overdoses should be notified using SOP 17 Recording and Reporting of Protocol Deviations and Violations, Serious Breaches of Protocol or GCP.
- Overdoses be observed from pill counts, diary cards, drug charts and patient comment.

- The affect of an overdose on the final analysis will determined on a case by case basis.
- If an SAE is associated with the overdose ensure the overdose will be fully described in the SAE report form.

13.9 Treatment Stopping Rules

The trial may be prematurely discontinued by the Sponsor, Chief Investigator or Regulatory Authority on the basis of new safety information or for other reasons given by the Data Monitoring & Ethics Committee / Trial Steering Committee regulatory authority or ethics committee concerned.

If the trial is prematurely discontinued, active participants will be informed and no further participant data will be collected. The Competent Authority and Research Ethics Committee will be informed within 15 days of the early termination of the trial.

13.10 Development safety update reports

UHSM SOP 7a Development Safety Update Reporting must be followed.

The CI or their delegate will submit DSURs once a year throughout the clinical trial, or on request to the Competent Authority (MHRA in the UK), Ethics Committee and Sponsor.

13.11 Signal review and trend analysis

13.11.1 Safety signal review

UHSM SOP 2c Safety monitoring for UHSM Sponsored CTIMPs must be followed.

13.11.2 Trend analysis of adverse events

UHSM SOP 2c Safety monitoring for UHSM Sponsored CTIMPs must be followed.

14 Finance

The trial is funded by a grant from ***Breast Cancer Now***:
Fifth Floor, Ibex House, 42-47 Minories,
London EC39 1DY
Switchboard 0333 207 0300
www.breastcancernow.org

15 Publication and dissemination

We will aim to publish this pilot study in a peer reviewed cancer prevention journal and present the data at national and international cancer/prevention conferences. This will be carried out following UHSM's SOP38 Publication of UHSM Sponsored Research**.** We will inform all study participants that they can request the results of the study.

16 Trial Governance

A formal independent data monitoring committee will not be convened as this trial evaluates the repurposing of an agent shown to be safe in this age group of women in randomised controlled phase III studies. However, there remains a need to examine the safety and procedural data from the trial at regular intervals. The trial management group will take on this role:

16.1 Trial Management Group

The Trial Management Group will be under the chairmanship of the Chief Investigator (SH). The TMG will meet formally at least every six months to review safety, recruitment and procedural data. A formal report will be produced every six months and a meeting of the TMG convened to discuss the findings. The minutes and conclusions of these meetings will be submitted to the Sponsor for review and to obtain the approval to continue with study procedures. The TMG will consist of the following clinical and laboratory investigators:

1. Chair - Dr Sacha Howell
2. Recruiting clinicians: Professor Gareth Evans and Professor Tony Howell
3. Consultant Radiologists: Dr Anthony Maxwell and Dr Yit Lim
4. Imaging specialists: Dr Susan Astley and Dr Elaine Harkness
5. Basic scientists: Dr Robert Clarke, Professor Charles Streuli, Dr Michael Sherratt, Dr Bruno Simoes (PDRA employed on the grant)
6. Research Nurses

To be quorate the TMG meeting needs to have at least 1 attendee from each of groups 1-6. The production of the 6 monthly reports in no way obviates the need for safety reporting detailed in section 9 and appendix 2.

17 Confidentiality

All data will be kept strictly confidential according to Good Clinical Practice (GCP) Guidelines. All identifiable data will be stored by the Manchester University NHS Foundation Trust in a secure fashion for 20 years in accordance with the ICH GCP. Source data for the trial are the consent form, family and menstrual history, breast cancer risk

assessment, blood test results, radiology results and toxicity assessments. Data derived from analysis of patients' breast tissue will be held in the CRUK Manchester Institute and The Stopford Building at the University of Manchester associated with the participants trial number only. No patient identifiable data will be stored at either of these sites.

18 Quality Control

Monitoring of this trial will be to ensure compliance with Good Clinical Practice and scientific integrity will be managed and oversight retained, by the monitor assigned to monitor this trial as per the study monitoring plan. Data will be evaluated for compliance with the protocol and accuracy in relation to source documents. The monitors will verify that the clinical trial is conducted and data are generated, documented and reported in compliance with the protocol, GCP and the applicable regulatory requirements.

- an updated training record must be maintained for each member of the research team and retention of GCP training certificates which must be less than 3 years old
- protocol compliance will be monitored as part of the monitoring plan.

19 Protocol compliance

A "serious breach" is a breach which is likely to effect to a significant degree –

(a) the safety or physical or mental integrity of the subjects of the trial; or

(b) the scientific value of the trial

- prospective, planned deviations or waivers to the protocol are not allowed under the UK regulations on Clinical Trials and must not be used e.g. it is not acceptable to enrol a subject if they do not meet the eligibility criteria or restrictions specified in the trial protocol
- accidental protocol deviations can happen at any time. They must be adequately documented on the relevant forms and reported to the Chief Investigator and Sponsor immediately -. refer to procedure in SOP17 Recording and Reporting of Protocol Deviations and Violations, Serious Breaches of Protocol or GCP deviations from the protocol which are found to frequently recur are not acceptable, will require immediate action and could potentially be classified as a serious breach.
- the sponsor will be notified immediately of any case where the above definition applies during the trial conduct phase - refer to procedure in SOP17 Recording and Reporting of Protocol Deviations and Violations, Serious Breaches of Protocol or GCP

20 Amendments

Any amendments must follow the procedure described in UHSM's SOP 16 Amendment approval.

The amendment history will be tracked using a log stored in the TMF.

21 Insurance / Indemnity

For the management of the research: Indemnity is provided through NHS schemes.
For the design of the research: Indemnity is provided through NHS schemes.
For the conduct of the research: Indemnity is provided through NHS schemes.

22 References

Alowami S, Troup S, Al-Haddad S, Kirkpatrick I, Watson PH. Mammographic density is related to stroma and stromal proteoglycan expression. Breast Cancer Res. 2003;5:R129-35.

Anderson GL, Limacher M, Assaf AR, Bassford T, Beresford SA, Black H, Bonds D, Brunner R, Brzyski R, Caan B, Chlebowski R, Curb D, Gass M, Hays J, Heiss G, Hendrix S, Howard BV, Hsia J, Hubbell A, Jackson R, Johnson KC, Judd H, Kotchen JM, Kuller L, LaCroix AZ, Lane D, Langer RD, Lasser N, Lewis CE, Manson J, Margolis K, Ockene J, O'Sullivan MJ, Phillips L, Prentice RL, Ritenbaugh C, Robbins J, Rossouw JE, Sarto G, Stefanick ML, Van Horn L, Wactawski-Wende J, Wallace R, Wassertheil-Smoller S; Women's Health Initiative Steering Committee. Effects of conjugated equine estrogen in postmenopausal women with hysterectomy: the Women's Health Initiative randomized controlled trial. JAMA. 2004;291:1701-12.

Amarosa AR, McKellop J, Klautau Leite AP, Moccaldi M, Clendenen TV, Babb JS, Zeleniuch-Jacquotte A, Moy L, Kim S Evaluation of the kinetic properties of background parenchymal enhancement throughout the phases of the menstrual cycle. Radiology. 2013;268:356-65.

Assi V, Warwick J, Cuzick J, Duffy SW. Clinical and epidemiological issues in mammographic density. Nat Rev Clin Oncol. 2011;9:33-40

Attardi BJ, Burgenson J, Hild SA, Reel JR and Blye RP. CDB-4124 and its putative monodemethylated metabolite, CDB-4453, are potent antiprogestins with reduced antiglucocorticoid activity: in vitro comparison to mifepristone and CDB-2914. Molecular and Cellular Endocrinology 2002;188:111–123

Bascom JL, Radisky DC, Koh E, Fata JE, Lo A, Mori H, Roosta N, Hirai Y, Bissell MJ. Epimorphin is a novel regulator of the progesterone receptor isoform-a. Cancer Res. 2013;73:5719-29.

Beral V; Million Women Study Collaborators. Breast cancer and hormone-replacement therapy in the Million Women Study. Lancet. 2003 Aug 9;362(9382):419-27.

Berg WA, Krebs TL, Campassi C, Magder LS, Sun CC. Evaluation of 14- and 11-gauge directional, vacuum-assisted biopsy probes and 14-gauge biopsy guns in a breast parenchymal model. Radiology 1997;205:203-8.

Bramley M, Clarke RB, Howell A, Evans DGR, Armer T, Baildam AD and Anderson E. Effects of oestrogens and anti-oestrogens on normal breast tissue from women bearing BRCA1 and BRCA2 mutations. British Journal of Cancer (2006) 94, 1021 – 1028.

Brisken C, Park S, Vass T, Lydon JP, O'malley BW & Weinberg RA (1998) A paracrine role for the epithelial progesterone receptor in mammary gland development. Proc Natl Acad Sci U S A, 95 (9), 5076-81.

Buist DSM, Aiello EJ, Miglioretti DL, White E. Mammographic breast density, dense area, and breast area differences by phase in the menstrual cycle. Cancer Epidemiol Biomarkers Prev 2006;15:2303-6.

Burbank F. Stereotactic breast biopsy: comparison of 14- and 11-gauge Mammotome probe performance and complication rates. Am Surg. 1997;63:988-95.

Chlebowski RT, Hendrix SL, Langer RD, Stefanick ML, Gass M, Lane D, Rodabough RJ, Gilligan MA, Cyr MG, Thomson CA, Khandekar J, Petrovitch H, McTiernan A; WHI Investigators. Influence of estrogen plus progestin on breast cancer and mammography in healthy postmenopausal women: the Women's Health Initiative Randomized Trial. JAMA. 2003 Jun 25;289(24):3243-53.

Chlebowski RT, Anderson GL, Gass M, Lane DS, Aragaki AK, Kuller LH, Manson JE, Stefanick ML, Ockene J, Sarto GE, Johnson KC, Wactawski-Wende J, Ravdin PM, Schenken R, Hendrix SL, Rajkovic A, Rohan TE, Yasmeen S, Prentice RL; WHI Investigators. Estrogen plus progestin and breast cancer incidence and mortality in postmenopausal women. JAMA. 2010 Oct 20;304(15):1684-92.

Chlebowski RT, Kuller LH, Prentice RL, Stefanick ML, Manson JE, Gass M, Aragaki AK, Ockene JK, Lane DS, Sarto GE, Rajkovic A, Schenken R, Hendrix SL, Ravdin PM, Rohan TE, Yasmeen S, Anderson G; WHI Investigators. Breast cancer after use of estrogen plus progestin in postmenopausal women. N Engl J Med. 2009;360:573-87

Cigler T, Richardson H, Yaffe MJ, Fabian CJ, Johnston D, Ingle JN, Nassif E, Brunner RL, Wood ME, Pater JL, Hu H, Qi S, Tu D, Goss PE. A randomized, placebo-controlled trial (NCIC CTG MAP.2) examining the effects of exemestane on mammographic breast density, bone density, markers of bone metabolism and serum lipid levels in postmenopausal women. Breast Cancer Res Treat. 2011;126;453-61.

Clarke RB, Spence K, Anderson E, Howell A, Okano H, Potten CS. A putative human breast stem cell population is enriched for steroid receptor-positive cells. Dev Biol. 2005;277:443-56

Couto E, Qureshi SA, Hofvind S, Hilsen M, Aase H, Skaane P, Vatten L, Ursin G. Hormone therapy use and mammographic density in postmenopausal Norwegian women. Breast Cancer Res Treat. 2012;132:297-305.

Crick SL, Yin FCP. Assessing micromechanical properties of cells with atomic force microscopy: importance of the contact point. Biomech Model Mechanobiol. 2007;6:199-210.

Donnez J, Tatarchuk TF, Bouchard P, Puscasiu L, Zakharenko NF, Ivanova T, Ugocsai G, Mara M, Jilla MP, Bestel E, Terrill P, Osterloh I, Loumaye E; PEARL I Study Group. Ulipristal acetate versus placebo for fibroid treatment before surgery. N Engl J Med. 2012a;366:409-20

Donnez J, Tomaszewski J, Vázquez F, Bouchard P, Lemieszczuk B, Baró F, Nouri K, Selvaggi L, Sodowski K, Bestel E, Terrill P, Osterloh I, Loumaye E; PEARL II Study Group. Ulipristal acetate versus leuprolide acetate for uterine fibroids. N Engl J Med. 2012b;366:421-32

Eirew P1, Stingl J, Eaves CJ. Quantitation of human mammary epithelial stem cells with in vivo regenerative properties using a subrenal capsule xenotransplantation assay. Nat Protoc. 2010;5:1945-56

Engman M, Skoog L, Soderqvist G and Gemzell-Danielsson K. The effect of mifepristone on breast cell proliferation in premenopausal women evaluated through fine needle aspiration cytology. Human Reproduction 2008;23;2072–2079

Evans DG, Harvie M, Bundred N, Howell A. Uptake of breast cancer prevention and screening trials. J Med Genet 2010;47:853-5

Ferguson JE, Schor AM, Howell A, Ferguson MW. Changes in the extracellular matrix of the normal human breast during the menstrual cycle. Cell Tissue Res. 1992;268:167-77

Ghosh K, Brandt KR, Reynolds C, Scott CG, Pankratz VS, Riehle DL, Lingle WL, Odogwu T, Radisky DC, Visscher DW, Ingle JN, Hartmann LC, Vachon CM. Tissue composition of mammographically dense and non-dense breast tissue. Breast Cancer Res Treat. 2012;131:267-75.

Golatta M, Schweitzer-Martin M, Harcos A, et al. Normal breast tissue stiffness measured by a new ultrasound technique: virtual touch tissue imaging quantification (VTIQ). Eur J Radiol 2013;82:e676–9.

Gonzalez-Suarez E, Allison P. Jacob, Jon Jones, Robert Miller, Martine P. Roudier-Meyer, Ryan Erwert, Jan Pinkas, Dan Branstetter & William C. Dougall

RANK ligand mediates progestin-induced mammary epithelial proliferation and carcinogenesis 2010 Nature;468:103-9

Graham JD, Mote PA, Salagame U, van Dijk JH, Balleine RL, Huschtscha LI, Reddel RR, Clarke CL DNA replication licensing and progenitor numbers are increased by progesterone in normal human breast. Endocrinology. 2009;150:3318-26

Graham HK, Hodson NW, Hoyland JA, Millward-Sadler SJ, Garrod D, Scothern A, et al. Tissue section AFM: In situ ultrastructural imaging of native biomolecules. Matrix Biol. 2010;29:254-60.

Graham HK, Akhtar R, Kridiotis C, Derby B, Kundu T, Trafford AW, et al. Localised micro-mechanical stiffening in the ageing aorta. Mech Ageing Dev. 2011;132:459-67.

Greendale GA, Reboussin BA, Slone S, Wasilauskas C, Pike MC, Ursin G. Postmenopausal hormone therapy and change in mammographic density. J Natl Cancer Inst. 2003;95:30-7

Guo YP, Martin LJ, Hanna W, Banerjee D, Miller N, Fishell E, Khokha R, Boyd NF. Growth factors and stromal matrix proteins associated with mammographic densities. Cancer Epidemiol Biomarkers Prev. 2001;10:243-8

Hopp, T. A., Weiss, H. L., Hilsenbeck, S. G., Cui, Y., Allred, D. C., Horwitz, K. B. & Fuqua, S. A. W. (2004) Breast Cancer Patients with Progesterone Receptor PR-A-Rich Tumors Have Poorer Disease-Free Survival Rates. Clinical Cancer Research, 10 (8), 2751-2760.

Hovhannisyan G, Chow L, Schlosser A, Yaffe MJ, Boyd NF, Martin LJ. Differences in measured mammographic density in the menstrual cycle. Cancer Epidemiol Biomarkers Prev. 2009;18:1993-9.

Jones LP, Li M, Halama ED, Ma Y, Lubet R, Grubbs CJ, Deng CX, Rosen EM, Furth PA. Promotion of mammary cancer development by tamoxifen in a mouse model of Brca1-mutation-related breast cancer. Oncogene. 2005 May 19;24(22):3554-62.

Joshi, P. A., Jackson, H. W., Beristain, A. G., Di Grappa, M. A., Mote, P. A., Clarke, C. L., Stingl, J., Waterhouse, P. D. & Khokha, R. (2010) Progesterone induces adult mammary stem cell expansion. Nature, 465 (7299), 803-7.

Jud SM, Häberle L, Fasching PA, et al. Correlates of mammographic density in B-mode ultrasound and real time elastography. Eur J Cancer Prev 2012;21:343–9.

Kajihara M, Goto M, Hirayama Y, Okunishi S, Kaoku S, Konishi E, Shinkura N.Effect of the menstrual cycle on background parenchymal enhancement in breast MR imaging. Magn Reson Med Sci. 2013;12:39-45

King V, Brooks JD, Bernstein JL, Reiner AS, Pike MC, Morris EA. Background parenchymal enhancement at breast MR imaging and breast cancer risk. Radiology. 2011;260:50-60.

Klijn, J. G., Setyono-Han, B. & Foekens, J. A. Progesterone antagonists and progesterone receptor modulators in the treatment of breast cancer. Steroids 2000;65:825-30.

Lee E, Ingles SA, Van Den Berg D, Wang W, Lavallee C, Huang MH, Crandall CJ, Stanczyk FZ, Greendale GA, Ursin G. Progestogen levels, progesterone receptor gene polymorphisms, and mammographic density changes: results from the Postmenopausal Estrogen/Progestin Interventions Mammographic Density Study. Menopause. 2012;19:302-10

Li T, Sun L, Miller N, Nicklee T, Woo J, Hulse-Smith L, Tsao MS, Khokha R, Martin L, Boyd N. The association of measured breast tissue characteristics with mammographic density and other risk factors for breast cancer. Cancer Epidemiol Biomarkers Prev. 2005;14:343-9

Lim E, Vaillant F, Wu D, Forrest NC, Pal B, Hart AH, Asselin-Labat ML, Gyorki DE, Ward T, Partanen A, Feleppa F, Huschtscha LI, Thorne HJ; kConFab, Fox SB, Yan M, French JD, Brown MA, Smyth GK, Visvader JE, Lindeman GJ. Aberrant luminal progenitors as the candidate target population for basal tumor development in BRCA1 mutation carriers. Nat Med. 2009 Aug;15(8):907-13.

Lydon, J. P., Demayo, F. J., Funk, C. R., Mani, S. K., Hughes, A. R., Montgomery, C. A., Jr., Shyamala, G., Conneely, O. M. & O'malley, B. W. (1995) Mice lacking progesterone receptor exhibit pleiotropic reproductive abnormalities. Genes Dev, 9 (18), 2266-78.

Maller O, Hansen KC, Lyons TR, Acerbi I, Weaver VM, Prekeris R, Tan AC, Schedin P. Collagen architecture in pregnancy-induced protection from breast cancer. J Cell Sci. 2013;126:4108-10.

McTiernan A, Martin CF, Peck JD, Aragaki AK, Chlebowski RT, Pisano ED, Wang CY, Brunner RL, Johnson KC, Manson JE, Lewis CE, Kotchen JM, Hulka BS; Women's Health Initiative Mammogram Density Study Investigators. Estrogen-plus-progestin use and mammographic density in postmenopausal women: Women's Health Initiative randomized trial. J Natl Cancer Inst. 2005 Sep 21;97(18):1366-76.

McTiernan A, Chlebowski RT, Martin C, Peck JD, Aragaki A, Pisano ED, Wang CY, Johnson KC, Manson JE, Wallace RB, Vitolins MZ, Heiss G. Conjugated equine estrogen influence on mammographic density in postmenopausal women in a substudy of the women's health initiative randomized trial. J Clin Oncol. 2009;27:6135-43.

Mohsin SK, Allred DC, Osborne CK, Cruz A, Otto P, Chew H, Clark GM, Elledge RM. Morphologic and immunophenotypic markers as surrogate endpoints of tamoxifen effect for prevention of breast cancer. Breast Cancer Res Treat. 2005;94:205-11.

Morrow M, Chatterton RT Jr, Rademaker AW, Hou N, Jordan VC, Hendrick RE, Khan SA. A prospective study of variability in mammographic density during the menstrual cycle. Breast Cancer Res Treat. 2010;121:565-74.

Mote, P. A., Bartow, S., Tran, N. & Clarke, C. L. Loss of Co-ordinate Expression of Progesterone Receptors A and B is an Early Event in Breast Carcinogenesis. Breast Cancer Research and Treatment, 2002; 72 (2), 163-172.

Mote, P. A., Leary, J. A., Avery, K. A., Sandelin, K., Chenevix-Trench, G., Kirk, J. A. & Clarke, C. L. (2004) Germ-line mutations in BRCA1 or BRCA2 in the normal breast are associated with altered expression of estrogen-responsive proteins and the predominance of progesterone receptor A. Genes, Chromosomes and Cancer, 39 (3), 236-248.

Mulac-Jericevic, B., Lydon, J. P., Demayo, F. J. & Conneely, O. M. (2003) Defective mammary gland morphogenesis in mice lacking the progesterone receptor B isoform. Proceedings of the National Academy of Sciences, 100 (17), 9744-9749.

Nallasamy S, K Jaeyeon, Sitruk-Ware R, Bagchi M, Bagchi I. Ulipristal Blocks Ovulation by Inhibiting Progesterone Receptor–Dependent Pathways Intrinsic to the Ovary. Reproductive Sciences 2012; 20:371-381

Navarrete MA, Maier CM, Falzoni R, Quadros LG, Lima GR, Baracat EC, Nazário AC Assessment of the proliferative, apoptotic and cellular renovation indices of the human mammary epithelium during the follicular and luteal phases of the menstrual cycle. Breast Cancer Res. 2005;7:R306-13.

Obr, A. E. & Edwards, D. P. The biology of progesterone receptor in the normal mammary gland and in breast cancer. Molecular and Cellular Endocrinology, 2012;357:4-17.

O'Flynn EAM, Morgan VA, Giles SL, et al. Diffusion weighted imaging of the normal breast: reproducibility of apparent diffusion coefficient measurements and variation with menstrual cycle and menopausal status. Eur Radiol 2012;22:1512–8.

Ong KR, Sims AH, Harvie M, Chapman M, Dunn WB, Broadhurst D, Goodacre R, Wilson M, Thomas N, Clarke RB, Howell A. Biomarkers of dietary energy restriction in women at increased risk of breast cancer.Cancer Prev Res (Phila). 2009;2:720-31

Pardo I, Lillemoe HA, Blosser RJ, Choi M, Sauder CA, Doxey DK, Mathieson T, Hancock BA, Baptiste D, Atale R, Hickenbotham M, Zhu J, Glasscock J, Storniolo AM, Zheng F, Doerge R, Liu Y, Badve S, Radovich M, Clare SE. Next-generation transcriptome sequencing of the premenopausal breast epithelium using specimens from a normal human breast tissue bank. Breast Cancer Res. 2014;16:R26

Partridge SC, McKinnon GC, Henry RG, et al. Menstrual cycle variation of apparent diffusion coefficients measured in the normal breast using MRI. J Magn Reson Imaging 2001;14:433–8

Pearman L, Kagan R, Arsenault J and Muram D. The effects of raloxifene on mammographic breast density: a review of clinical trials. Menopause 2010;17: 654-659

Pike MC, Spicer DV, Dahmoush L, Press MF. Review Estrogens, progestogens, normal breast cell proliferation, and breast cancer risk. Epidemiol Rev. 1993; 15(1):17-35

Pike MC, Pearce CL. Mammographic density, MRI background parenchymal enhancement and breast cancer risk. Ann Oncol. 2013;24:viii37-viii41.

Pohl O, Harvey PW, McKeag S, Boley SE, Gotteland JP. Carcinogenicity and chronic rodent toxicity of the selective progesterone receptor modulator ulipristal acetate. Curr Drug Safety. 2013;8:77-97.

Poole AJ, Li Y, Kim Y, Lin SJ, Lee W, Lee EYP. Prevention of Brca1-Mediated Mammary Tumorigenesis in Mice by a Progesterone Antagonist. Science 2006;314:1467-70

Potten CS, Watson RJ, Williams GT, Tickle S, Roberts SA, Harris M, Howell A. The effect of age and menstrual cycle upon proliferative activity of the normal human breast. Br J Cancer. 1988;58:163-70.

Rzymski P, Skórzewska A, Skibińska-Zielińska M, et al. Factors influencing breast elasticity measured by the ultrasound Shear Wave elastography - preliminary results. Arch Med Sci 2011;7:127–33.

Scaranelo AM, Carrillo MC, Fleming R, Jacks LM, Kulkarni SR, Crystal P. Pilot study of quantitative analysis of background enhancement on breast MR images: association with menstrual cycle and mammographic breast density. Radiology. 2013;267:692-700.

Schramek D, Andreas Leibbrandt, Verena Sigl, Lukas Kenner, John A. Pospisilik, Heather J. Lee, Reiko Hanada, Purna A. Joshi, Antonios Aliprantis, Laurie Glimcher, Manolis Pasparakis, Rama Khokha, Christopher J. Ormandy, Martin Widschwendter, Georg Schett & Josef M. Penninger. Osteoclast differentiation factor RANKL controls development of progestin-driven mammary cancer. 2010 Nature;468:98-103

Simian M, Bissell MJ, Barcellos-Hoff MH, Shyamala G. Estrogen and progesterone receptors have distinct roles in the establishment of the hyperplastic phenotype in PR-A transgenic mice. Breast Cancer Res. 2009;11:R72.

Ursin G, Parisky YR, Pike MC, Spicer DV. Mammographic density changes during the menstrual cycle. Cancer Epidemiol Biomarkers Prev 2001;10:141 – 2.

Wang J, Azziz A, Fan B, Malkov S, Klifa C, Newitt D, Yitta S, Hylton N, Kerlikowske K, Shepherd JA. Agreement of mammographic measures of volumetric breast density to MRI. PLoS One. 2013;8:e81653.

Widschwendter M, Rosenthal AN, Philpott S, Rizzuto I, Fraser L, Hayward J, Intermaggio MP, Edlund CK, Ramus SJ, Gayther SA, Dubeau L, Fourkala EO, Zaikin A, Menon U, Jacobs IJ. The sex hormone system in carriers of BRCA1/2 mutations: a case-control study. Lancet Oncol. 2013;14:1226-32

Yang WT, Lewis MT, Hess K, Wong H, Tsimelzon A, Karadag N, Cairo M, Wei C, Meric-Bernstam F, Brown P, Arun B, Hortobagyi GN, Sahin A, Chang JC.Decreased TGFbeta signaling and increased COX2 expression in high risk women with increased mammographic breast density. Breast Cancer Res Treat. 2010;119:305-14.

Appendix 1: inhibitors/inducers of CYP3A4 not to be taken with UA

Potent inhibitors of CYP3A4 that should not be taken with ulipristal acetate (summary of product characteristics 'not recommended')

ketoconazole, Itraconazole

ritonavir, Indanavir, Saquinavir, Nelfinavir

nefazodone

telithromycin, clarithromycin

Nefazodone

Moderate inhibitors of CYP3A4 that should not be taken with ulipristal acetate (summary of product characteristics 'not recommended')

Erythromycin

verapamil

grapefruit juice (Patients should abstain from eating large amounts of grapefruit and Seville oranges (and other products containing these fruits eg, grapefruit juice or marmalade) during the study (e.g., no more than a small glass of grapefruit juice (120 mL) or half a grapefruit or 1-2 teaspoons (15 g) of Seville orange marmalade daily)

Potent inducers of CYP3A4 that should not be taken with ulipristal acetate (summary of product characteristics 'not recommended')

Rifampicin, Rifabutin

Carbamazepine, Oxcarbazepine

phenytoin, fosphenytoin,

phenobarbital

primidone

St John´s wort

Efavirenz, nevirapine, ritonavir

If any of the above moderate or potent inhibitors or potent inducers of CYP3A4 are absolutely required during the study then the trial treatment (ulipristal acetate) must be discontinued.

P-glycoprotein (P-gP) substrates

UA may be an inhibitor of P-gP at clinically relevant concentrations in the gastrointestinal wall during absorption. Simultaneous administration of UA and a P-gp substrate has not been studied and an interaction cannot be excluded. In vivo results show that UA (single 10 mg tablet) 1.5 hour before administration of the P-gP substrate fexofenadine (60 mg) has no clinically relevant effects on the pharmacokinetic of fexofenadine.

It is therefore recommended that co-administration of UA and P-gP substrates (e.g. digoxin and fexofenadine) should be separated in time by at least 1.5 hours.

Appendix 2: Data Entry into Excel Spreadsheets and Statistical Software Programmes and QC checks

Data Entry into Excel Spreadsheets

Data will be taken from the paper CRF. Data may be entered at the end of the study in its entirety or in batches. If data will be entered in batches, a new version of the database must be resaved and dated each time. Data entry should be performed by suitably trained and delegated staff using the 'single data entry with control checks' method.

Single Data Entry – Data is entered into the spreadsheet or database and then a visual check is performed between what is recorded on the paper source documents and what was entered into the spreadsheet or database.

The procedure is as follows:

1. Data is entered directly from the CRF into the spreadsheet ensuring all required fields are completed and in the required format e.g. correct number of decimal places.

2. Highlight all of the data that has been entered and click 'format', and ensure 'lock cell' is selected.

3. Go to review, protect sheet, and add a study specific password. This procedure will allow the data that has been entered to be locked whilst Quality Control (QC) is completed whilst still allowing further data to be added (if required) at a later date.

4. Save the excel spreadsheet using the agreed format, which identifies the file status, version and date corresponding to the data entry. This should be saved in the study specific ISF section for data management.

5. Print the spreadsheet with formulae (if applicable) unhidden. The print out must be signed and dated by the person who performed the data entry and then passed to the member of the research team who willl perform the QC .

6. The data will then be QC checked as per the procedure below

QC check of Single Data Entry

On each occasion data is entered and printed, the data transcriptions must be 100% checked for accuracy and any errors highlighted for correction. The QC check must be performed by a second trained staff member who is not the person who originally entered the data.

The checking process should ensure, where appropriate the following:

- The correct spreadsheet has been used.
- There are no typographical errors.
- The correct study title and protocol number (if required) and document version have been included.

- The correct subjects, time points, treatment periods have been included.
- All data points have been entered correctly.
- The correct number of decimal places has been used.
- All repeat measures have been checked and the correct values included.
- If a spreadsheet contains formulae, it should be printed after the formula has been entered and manually checked. The electronic calculation is checked against the manual calculation. If errors are identified in the formulae, the amendments are made, the sheet reprinted and independently re-checked.
- Each cell of the printed spreadsheet must be checked against the source data and the QC checker must tick by hand each cell that is correct and highlight by hand each cell that is incorrect.
- On completion of the QC check, the sheet is signed, dated by the QC checker and returned to the member of data entry staff for correction of the highlighted cells if discrepancies are found.
- Each printed page may be checked, signed and dated the day the check is performed. If however the check is performed on different days the signature and the date should reflect this.
- The spreadsheet should be corrected as detailed above and then resaved, reprinted and rechecked until no errors exist. Each printing and QC check will be signed and dated by the member of staff performing this action.

- The process is repeated until the final spreadsheet has been completed, QC checked and labelled as 'FINAL' and dated. The final signed spreadsheet must be saved as a PDF and filed in the paper and electronic study ISF and then transferred to the investigator, statistician or data Management Company depending on the specific study.

7. To add further data to the password protected excel spreadsheet or to amend data following the QC check, the following procedure should be followed:

8. Go to review, unprotect sheet and add the password.

9. Enter the next batch of data directly from the CRF or amend data previously added (following QC review) ensuring all fields are completed.

10. On completion of the data entry session, select the data you wish to protect, click 'format', and then 'lock cell'.

11. Go to review, protect sheet, and add the study specific password.

12. Save the excel spreadsheet using the agreed format and which identifies the file status, new version and date corresponding to the data entry. This should be saved in the study specific folder.

13. Move the superseded version of the excel spreadsheet into a folder named 'superseded'.

14. Print the spreadsheet with the formulae unhidden (if applicable). Then the spreadsheet must be signed and dated by the person who performed the data entry and then passed to the member of the research team who willl perform the QC

Data validation and amendments

A member of staff, authorised by the investigator, should validate the data against pre-defined ranges and logical linked responses. Any discrepancies found should be checked by a second staff member who will refer back to the source data. Any data amendment required will be carried out by this individual. This QC check will be signed and dated as per the usual convention.

Data Lock

After all QC checks have been completed, a database lock will be performed and a 'read-only' copy will be retained. Appropriate documentation of the timing of datalock will be made.

Transfer of Data to a Statistical Software Programme

Data may be imported directly or copied and pasted from the excel spreadsheet into a programme for statistical analysis e.g. SPSS, once data entry is complete.

If copied and pasted into SPSS:

1. The SPSS database should be formatted with required fields prior to any data transfer.

2. Where possible, the spreadsheet must be copied as a whole rather than as individual cells or rows.

3. Select all data by clicking in the left hand corner. Select 'edit' and then 'copy'.

4. Paste the data into the SPSS database.

A random 10% QC check of the SPSS database must be completed to ensure an accurate transfer. Once this is complete, the SPSS file may be printed and hand signed and dated to state that the check was completed and filed in the ISF. From this point onwards, the investigator or statistician is responsible for maintaining and manipulating of the SPSS file whilst carrying out statistical analysis to ensure the data remains unchanged. The original excel file should still be printed, signed, filed and retained.

Appendix 3: A guide to performing causality assessments

The following factors should be considered when deciding if there is a "reasonable possibility" that an SAE may have been caused by the drug.

- Time course. Exposure to suspect drug. Has the subject actually received the suspect drug? Did the SAE occur in a reasonable temporal relationship to the administration of the suspect drug?

- Consistency with known drug profile. Was the AE consistent with the previous knowledge of the suspect drug (pharmacology and toxicology) or drugs of the same pharmacological class? Or could the SAE be anticipated from its pharmacological properties?

- De-challenge experience. Did the SAE resolve or improve on stopping or reducing the dose of the suspect drug?

- No alternative cause. The SAE cannot be reasonably explained by another aetiology such as the underlying disease, other drugs, other host or environmental factors.

- Laboratory tests. A specific laboratory investigation (if performed) has confirmed the relationship?

A "reasonable possibility" could be considered to exist for an SAE where one or more of these factors exist.

In contrast there would not be a "reasonable possibility" of causality if none of the above criteria apply, or where there is evidence of exposure and a reasonable time course, but any de-challenge (if performed) is negative or ambiguous or there is another more likely cause of the SAE.

In difficult cases other factors should be considered such as:

- Is this a recognised feature of overdose of the drug?
- Is there a known mechanism?

Ambiguous cases should be considered as being a "reasonable possibility" of causal relationship unless further evidence becomes available to refute this. Causal relationship in cases where the disease under study has deteriorated due to lack of effect should be classified as no reasonable possibility.

Appendix 4: SAE reporting procedures

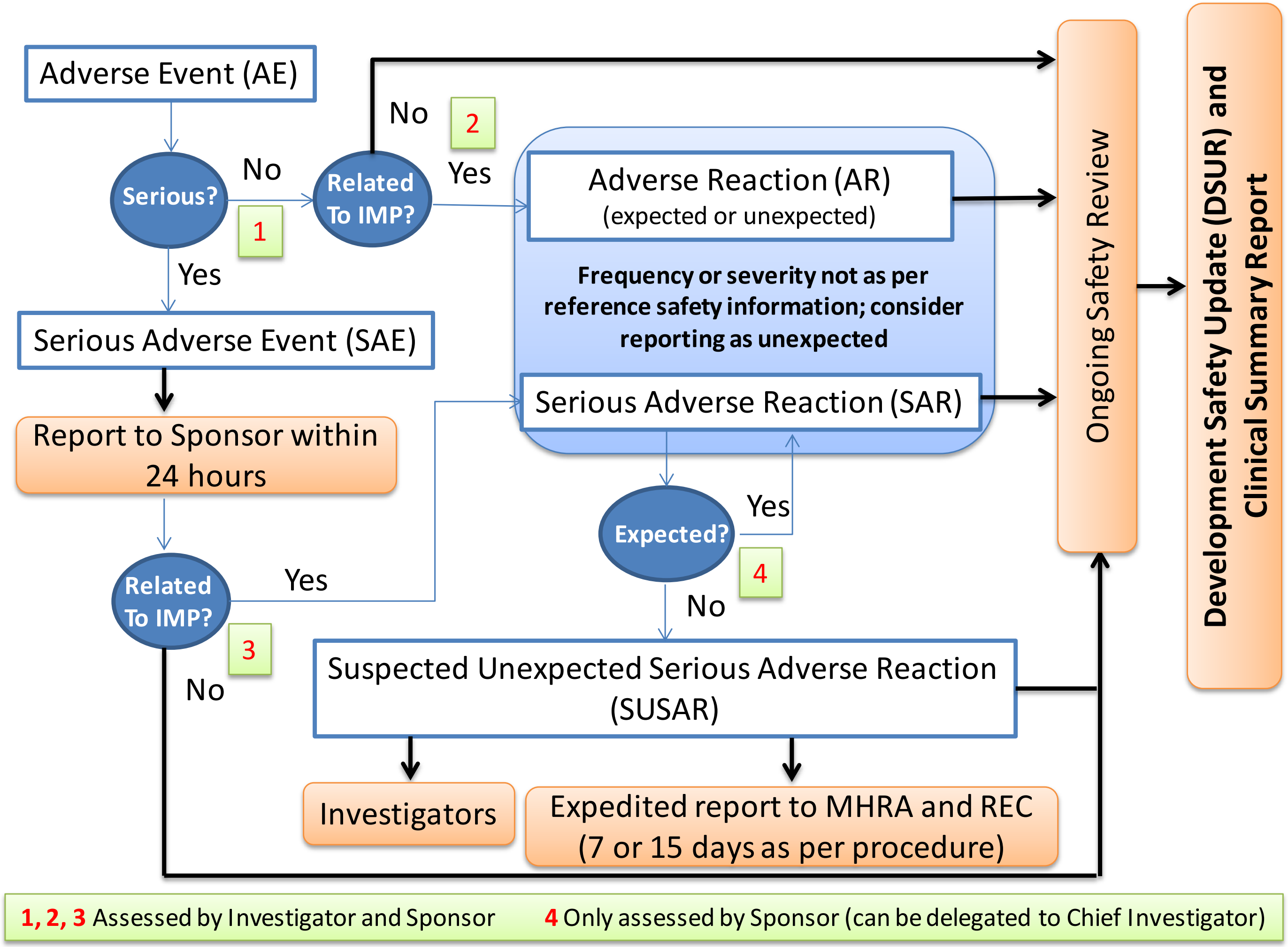

Appendix 5: Summary of trial procedures

Time	Day -7 EOM +/- 4 days	Day -7 EOM +/- 4 days to OM	Telephone call to patient regarding histology results and onset of menses	Day 29 +4 /- 7 days (Week 5)	Day 57 +4 /- 7 days (Week 9)	Day 78-85 (Week 12-13)	Day 113 +/- 4 days (week 17)
Cycles of treatment			Cycle 1 D1	Cycle 2 D1	Cycle 3 D1		
Demographic information, medical history and baseline symptoms documented	X						
Weight recorded	X			X	X	X	X
Height recorded	X						
Concomitant medications recorded	X			X	X		
Serum progesterone	X					X	
Serum βhCG pregnancy test	X						
APTT/PT/FBC (for Hb and platelets)/U+E (for serum creatinine)/LFT (for bilirubin, ALT, ALP and LDH)	X			X****	X****	X	X****
Lipid profile (for total cholesterol)	X					X	
TFT (for TSH and T4)	X					X	
Serum and Plasma samples for storage	X					X	
MRI		X*				X	
US guided VAB including breast electrical impedance		X*				X***	
H&E pathological analysis of breast tissue from VAB to exclude invasive cancer and DCIS		X**					
Symptom review				X	X	X	X
Document AEs		X		X	X	X	X
Urine pregnancy test				X	X	X	
Dispense ulipristal acetate 5mg daily: 3 x 28 day packs		X					
Patient to start supply of dispensed UA			X				
Drug accountability check				X	X	X	

*MRI to be performed before VAB. Contraindications to MRI, such as intracranial aneurysm clips, implanted electrical devices and intra-ocular metallic foreign bodies, will exclude participants from undergoing an MRI. In addition, should a participant not fulfil the size/weight requirements of the scanner or if the scanner breaks down resulting in a participant not having an MRI scan they can continue with other trial procedures.**H&E staining and analysis to be performed using part of the tissue from the VAB but before the study treatment commences. *** or prophylactic mastectomy. APTT activated Partial Thromboplastin Time, PT Prothrombin Time, FBC Full Blood Count, U+E Urea and Electrolytes, LFTs liver function tests. ****only LFTs will be measured.
