# Supplementary material for: Anti-progestin therapy targets hallmarks of breast cancer risk
Source: Nature. 2025 Nov 5;648(8094):736–45. doi: 10.1038/s41586-025-09684-7 (PMC12711567; doi:10.1038/s41586-025-09684-7)
Supplement: Supplementary file 2 — Reporting Summary [file 41586_2025_9684_MOESM2_ESM.pdf]

nature portfolio

Corresponding author(s): Bruno M Simões, Robert B Clarke, Sacha J Howell

Last updated by author(s): Sep 22, 2025

Reporting Summary

Nature Portfolio wishes to improve the reproducibility of the work that we publish. This form provides structure for consistency and transparency in reporting. For further information on Nature Portfolio policies, see our Editorial Policies and the Editorial Policy Checklist.

Statistics

For all statistical analyses, confirm that the following items are present in the figure legend, table legend, main text, or Methods section.

n/a	Confirmed	
☐	☒	The exact sample size (n) for each experimental group/condition, given as a discrete number and unit of measurement
☐	☒	A statement on whether measurements were taken from distinct samples or whether the same sample was measured repeatedly
☐	☒	The statistical test(s) used AND whether they are one- or two-sided<br>*Only common tests should be described solely by name; describe more complex techniques in the Methods section.*
☐	☒	A description of all covariates tested
☐	☒	A description of any assumptions or corrections, such as tests of normality and adjustment for multiple comparisons
☐	☒	A full description of the statistical parameters including central tendency (e.g. means) or other basic estimates (e.g. regression coefficient) AND variation (e.g. standard deviation) or associated estimates of uncertainty (e.g. confidence intervals)
☐	☒	For null hypothesis testing, the test statistic (e.g. F, t, r) with confidence intervals, effect sizes, degrees of freedom and P value noted<br>*Give P values as exact values whenever suitable.*
☒	☐	For Bayesian analysis, information on the choice of priors and Markov chain Monte Carlo settings
☒	☐	For hierarchical and complex designs, identification of the appropriate level for tests and full reporting of outcomes
☒	☐	Estimates of effect sizes (e.g. Cohen's d, Pearson's r), indicating how they were calculated

Our web collection on statistics for biologists contains articles on many of the points above.

Software and code

Policy information about availability of computer code

Data collection	Tissue bulk RNAseq: NextSeq 500 sequencer (Illumina), Nextflow (v19.10.0), nf-core/rnaseq (v1.3) using GRCh38 human genome as reference<br>scRNAseq: NovaSeq 6000 (Illumina), CellRanger (3.02) using GRCh38 human genome as reference<br><br>Mass Spectrometry: UltiMate 3000 Rapid Separation LC system (RSLC, Dionex Corporation) coupled to a Q Exactive HF™ mass spectrometer (Thermo Fisher), MaxQuant (v1.6.14.0)<br><br>Imaging Mass Cytometry: Hyperion Imaging System (Standard BioTools Inc., Fluidigm CyTOF Software v7.0)
Data analysis	All custom code and scripts used to generate analyses of the tissue bulk RNAseq, the single-cell RNA-seq, and the IMC data are available at the following link: https://zenodo.org/records/11369094<br><br>Other software packages include:<br><br>Tissue bulk RNAseq: Nextflow (v19.10.0); nf-core/rnaseq (v1.3) using GRCh38 human genome as reference; Ensembl BioMart (v101); DESeq2 (v1.26.0); pheatmap (v1.0.12); stats (v3.6.0), SummarisedExperiment (v1.16.1)<br><br>scRNAseq: DropletUtils (v1.10.3), scuttle (v1.18.6), org.Hs.eg.db (v3.12.0), batchelor (v1.6.3), SNN graph (scran v1.18.7), igraph (v1.2.6), scater (v1.18.6), Cell Chat (v2.1.2), NicheNet (v2.2.0), Memento (v0.1.0), UpsetR (v1.4.0), gprofiler2 (v0.2.3), python (v3.10.13), scanpy (v1.9.6)<br><br>Mass Spectrometry: MSqRob, clusterProfiler (v4.6.0), Reactome pathway (v.65)

Flow cytometry: FACSDIVA (v8.0)

Histology: Aperio ImageScope Digital Pathology Slide viewer (v12.4.6), HALO Image Analysis Software (Indica Labs, v3.6.4134.314)

MRI scans: software ITK-SNP (v3.8.0)

Mammographic Density: Volpara density (v1.5.0)

Imaging Mass Cytometry: Steinbock (v0.15.0), MATLAB (v2022b)

Real-time PCR: QuantStudio Design and Analysis software (v2.6.0)

Western blot: Image Lab (v6.1, BioRad)

For manuscripts utilizing custom algorithms or software that are central to the research but not yet described in published literature, software must be made available to editors and reviewers. We strongly encourage code deposition in a community repository (e.g. GitHub). See the Nature Portfolio guidelines for submitting code & software for further information.

Data

Policy information about availability of data

All manuscripts must include a data availability statement. This statement should provide the following information, where applicable:

- Accession codes, unique identifiers, or web links for publicly available datasets
- A description of any restrictions on data availability
- For clinical datasets or third party data, please ensure that the statement adheres to our policy

Datasets generated in the BC-APPS1 study:

All bulk and single cell RNA-sequencing data has been deposited in the Array Express data base (https://www.ebi.ac.uk/biostudies/arrayexpress) and can be retrieved by the following access IDs: E-MTAB-13720 (bulk RNAseq) and E-MTAB-13819 (scRNAseq).

The mass spectrometry proteomics data have been deposited to the ProteomeXchange Consortium via the PRIDE partner repository (https://www.ebi.ac.uk/pride/) with the dataset identifier PXD067122.

Datasets sourced from previously published studies:

RNAseq sequencing reads were aligned using the GRCh38 human genome as reference, available at https://www.ncbi.nlm.nih.gov/datasets/genome/GCF_000001405.39/

scRNAseq was compared to the scRNAseq dataset published by Reed et al, 2024 (doi: : 10.1038/s41588-024-01688-9; processed scRNAseq data can be downloaded at the CellXGene site - https://cellxgene.cziscience.com/collections/cd9a09e2- b440-4887-9163-6f8c684c7ced).

To assign peptides to protein groups, peptides were searched against the UniProt human proteome reference database (UP000005640) available at https://www.uniprot.org/proteomes/UP000005640

Proteins were classified as belonging to the "Matrisome" (structural ECM or ECM modifying) by searching against MatrisomeDB, a curated database of ECM proteins, available at https://sites.google.com/uic.edu/matrisome/matrisome-annotations/homo-sapiens

GSEA was performed using the Reactome Pathways database (v65), available at https://reactome.org/

Research involving human participants, their data, or biological material

Policy information about studies with human participants or human data. See also policy information about sex, gender (identity/presentation), and sexual orientation and race, ethnicity and racism.

Reporting on sex and gender	Only women (female sex) were eligible for this study.
Reporting on race, ethnicity, or other socially relevant groupings	Case selection was not based on any racial, ethnic or other demographic factors. Such data were not collected prospectively and are not reported in the manuscript.
Population characteristics	Patient demographics are presented in Supplementary Table 1.
Recruitment	All participants were recruited from the Family History Risk and Prevention Clinic at the Nightingale Centre, Wythenshawe Hospital, Manchester, UK. All participants were selected on their age (25–45 years), premenopausal status (including occurrence of regular menstrual cycles), absence of prior bilateral risk-reducing mastectomies, and having at least a moderately increased risk of breast cancer by virtue of a family history of the disease. Complete eligibility criteria are provided in the protocol (Supplementary Appendix 1). All eligible individuals were sent a letter of invitation and asked to contact the study team if interested in participating. Those who responded positively were provided with the participant information sheet and subsequently invited to clinic for consent, eligibility confirmation and recruitment to the study. As this was an investigational study for a drug with no proven benefit, requiring bilateral breast biopsies and two contrast-enhanced MRI scans, the participants were altruistic young women highly motivated to contribute to medical research. While this may

	reflect a degree of self-selection bias, there is no evidence to suggest this would influence objective molecular or radiological responses to the anti-progestin. Ethnicity and socioeconomic status of the participants were not recorded, and we therefore cannot comment on selection bias from those perspectives. Results may not, therefore, be generalisable to a broader population.
Ethics oversight	The Breast Cancer - Anti-Progestin Prevention Study 1 (BC-APPS1) was a single arm single centre phase II study registered under the name “A pilot prevention study of the effects of the anti- progestin Ulipristal Acetate (UA) on surrogate markers of breast cancer risk” (EudraCT registration number: 2015-001587-19; registration date: 15/07/2015; Greater Manchester – South, Research Ethics Committee number 15/NW/0478).

Note that full information on the approval of the study protocol must also be provided in the manuscript.

Field-specific reporting

Please select the one below that is the best fit for your research. If you are not sure, read the appropriate sections before making your selection.

☒ Life sciences ☐ Behavioural & social sciences ☐ Ecological, evolutionary & environmental sciences

For a reference copy of the document with all sections, see nature.com/documents/nr-reporting-summary-flat.pdf

Life sciences study design

All studies must disclose on these points even when the disclosure is negative.

Sample size	The planned sample size was n=30. This was based on a prior study using an alternate anti-progestin (mifepristone) which demonstrated significant reduction in Ki67 (the primary endpoint in our study) in 8 patients with no difference in 6 placebo treated women (Engman, M., et al, DOI: 10.1093/humrep/den228). A 30-subject study was proposed to provide sufficient data points to explore variability in response across both primary and secondary endpoints. Ultimately, 26 women were recruited due to a change in licensing for the drug by the MHRA. The objective for all experiments described below was to detect biological signals of ulipristal acetate activity in the breast. No formal sample size calculations were performed but all available samples were used, except for LCM proteomics, AFM, and IMC experiments where costs limited analyses. In those cases, a minimum of 4 paired samples were analysed.<br><br>Sample Prioritisation for Live Cell Analyses: As cell yield varied between participants due to differences in tissue composition, single-cell suspensions were prioritised as follows: (1) mammosphere formation efficiency assays (n = 19 pairs), (2) 2D colony-forming assays (n = 18 pairs), (3) flow cytometry (FACS) with cryopreserved cells (n = 17 pairs), and (4) scRNAseq, performed only on samples with ≥300,000 cryopreserved cells at both baseline and post-treatment (n = 6 pairs). This prioritisation ensured consistent use of available material across assays.<br><br>Sample Prioritisation for FFPE Analyses: Analyses were conducted as follows: (1) Ki67 immunostaining (n = 24 pairs), (2) tissue morphometry on samples with ≥3 well-defined lobules each containing ≥10 acini (n = 19 pairs), (3) PSR staining for collagen quantification on samples with ≥3 well-defined lobules (n = 22 pairs), (4) LCM proteomics, limited to 4 pairs with high epithelial content due to cost and processing constraints, and (5) IMC (Hyperion) imaging, using the same 4 pairs plus an additional 4 high-epithelium-content pairs (n = 8 total) as a confirmatory cohort.<br><br>Sample Prioritisation for Snap-Frozen Tissue Analyses: Snap-frozen cores were used for: (1) total RNA extraction for bulk RNA sequencing (n = 10 pairs, selected based on RNA quality), and (2) atomic force microscopy (AFM) stiffness measurements in samples showing ≥10% reduction in PSR staining post-treatment (n = 4 pairs).
Data exclusions	Analyses of paired breast tissue samples were applied to selected samples depending on epithelial cell availability, the technology utilized and its feasibility requirements. Single cell RNAseq data was excluded based on filtering and QC criteria as outlined in the methods section.
Replication	No technical replicates were performed on the same tissue samples; however, paired biopsy samples collected before and after treatment were analysed, representing biological replicates. All experiments included a minimum of four paired samples (i.e., four biological replicates). The replication attempts using these biological replicates were successful and the individual results across experiments are presented in the manuscript. For reference, bulk RNA sequencing was performed on only 10 paired samples, as detailed above. In the case of mammosphere formation efficiency assays, two baseline samples contained sufficient cells for plating but did not form any mammospheres.
Randomization	Previous studies have shown ulipristal acetate to suppress menstruation and endogenous progesterone levels in the majority of women (Donnez J. et al, DOI: 10.1056/NEJMoa1103182). A placebo arm was therefore not appropriate, as it would have been abundantly clear which women were taking ulipristal acetate. Randomisation between the established preventive agent tamoxifen and ulipristal acetate would have been another option; however, as both drugs reduce proliferation in the normal breast, the sample size required would have been very large and beyond the scope of a single institution study. A single-arm phase 2 study with paired biopsies was indicated in order to determine the effects of ulipristal acetate in women at increased breast cancer risk, with the potential for a follow-on randomised multicentre trial thereafter.
Blinding	Blinding was not applicable in this study, as all participants received the same treatment (ulipristal acetate). There was therefore no comparator group and, consequently, no need for participant or investigator blinding.

Reporting for specific materials, systems and methods

We require information from authors about some types of materials, experimental systems and methods used in many studies. Here, indicate whether each material, system or method listed is relevant to your study. If you are not sure if a list item applies to your research, read the appropriate section before selecting a response.

Materials & experimental systems

n/a	Involved in the study
☐	☒ Antibodies
☐	☒ Eukaryotic cell lines
☒	☐ Palaeontology and archaeology
☒	☐ Animals and other organisms
☐	☒ Clinical data
☒	☐ Dual use research of concern
☒	☐ Plants

Methods

n/a	Involved in the study
☒	☐ ChIP-seq
☐	☒ Flow cytometry
☒	☐ MRI-based neuroimaging

Antibodies

Antibodies used

Immunohistochemistry: Confirm Anti-Ki-67 (30-9) antibody (Roche,Ventana 790-4286);

Immunofluorescence: anti-SOX9 (Millipore, AB5535, 1:2000) and Ki67 (Dako, M 7240, 1:100);

Flow cytometry: anti-CD31 biotin-conjugated (eBioscience, 13-0319-82, 0.25 μg/ml), anti-CD45 biotin-conjugated (BioLegend, 304004, 0.125 μg/ml), APC-Cy7 streptavidin-conjugated (BioLegend, 405208, 0.2 μg/ml), anti-CD49f-APC (BioLegend, 313616, 0.5 μg/ml) and anti-EpCAM-FITC (StemCell Technologies, 10109, 1:5);

Imaging Mass Cytometry: anti-Alpha-Smooth muscle actin Pr-metal conjugated (#201508, Standard BioTools Inc., 1:1000), anti-SOX9 Sm-metal conjugated (#3147022D, Standard BioTools Inc., 1:75), anti-Fibronectin Sm-metal conjugated (#ab23750, Abcam, 1:75), anti-E-Cadherin Gd-metal conjugated (#201508, Standard BioTools Inc., 1:600), anti-Collagen VI Gd-metal conjugated (#ab6588, Abcam, 1:75), anti-Ki67 Er-metal conjugated (#201508, Standard BioTools Inc., 1:100) and anti-Collagen I Tm-metal conjugated (#201508, Standard BioTools Inc., 1:400);

Western blot: anti-SOX9 rabbit polyAb (#AB5535, Sigma, 1:1000), anti-C-KIT mouse polyAb (#MAB332, R&D Biosystems, 1:1000), anti-β-actin mouse mAb (#A1978, Sigma, 1:5000), goat anti-rabbit (#41424306, Dako, 1:5000) and goat anti-mouse (#41424131, Dako, 1:5000).

Validation

The commercial antibody validation can be found on each company's product sheet associated with the catalogue numbers. Additional validation details are summarised below.

Immunohistochemistry:
• Anti-Ki-67 (Roche/Ventana) – Manufacturer validation includes routine staining tests for sensitivity, specificity and precision on control tissues (lymph node, tonsil). Precision studies demonstrated between-lot reproducibility.

Immunofluorescence:
• Anti-SOX9 (Millipore) – Validated by Western blot in HepG2 lysates; control staining demonstrated in embryonic tissue and adult chondrocytes. Affinity-purified, reactive with human, mouse, rat, and chicken; widely cited (>1300 publications).
• Anti-Ki67 (Dako) – Extensively validated and cited (>4000 publications). Specific for Ki-67 with high lot-to-lot consistency, demonstrated specificity by Western blot and competitive binding assays. Control staining demonstrated in tonsillar sections.

Flow Cytometry:
• Anti-CD31 biotin-conjugated (eBioscience) – Validated for flow cytometry; tested on normal human peripheral blood cells.
• Anti-CD45 biotin-conjugated (BioLegend) – Each lot QC-tested by flow cytometry.
• Streptavidin-APC-Cy7 conjugate (BioLegend) – Each lot QC-tested by flow cytometry.
• Anti-CD49f-APC (BioLegend) – Each lot QC-tested by flow cytometry.
• Anti-EpCAM-FITC (StemCell Technologies) – Verified for flow cytometry applications.

Imaging Mass Cytometry:
• Anti-α-Smooth Muscle Actin (Standard BioTools Inc.) – Pathologist-verified for IMC on FFPE and frozen human tissue; QC-tested by IMC per lot; reactive with human and mouse.
• Anti-SOX9 (Standard BioTools Inc.) – Pathologist-verified for IMC on human FFPE tissue; QC-tested per lot; reactive with human, mouse, and rat.
• Anti-Fibronectin (Abcam) – Validated for IHC; >200 citations; strong specificity for human fibronectin.
• Anti-E-Cadherin (Standard BioTools Inc.) – Pathologist-verified for IMC on FFPE and frozen human tissue; QC-tested per lot; reactive with human, mouse, bovine.
• Anti-Collagen VI (Abcam) – Validated for Western blot on human tissue; strong specificity for type VI collagen, minimal cross-reactivity with other collagens; >150 citations.
• Anti-Ki-67 (Standard BioTools Inc.) – Pathologist-verified for IMC on FFPE and frozen human tissue; QC-tested per lot; reactive with human, mouse, rat, porcine.
• Anti-Collagen I (Standard BioTools Inc.) – Pathologist-verified for IMC on FFPE and frozen human tissue; QC-tested per lot; reactive with human and mouse.

Western Blot:

• SOX9 (Sigma/Merck) – Widely cited (>1300 publications); species reactivity includes human, mouse, rat.
• c-KIT (R&D Systems) – Detects human c-KIT in ELISA and WB; no cross-reactivity with mouse reported.
• β-actin (Sigma) – Validated for WB using extracts from human foreskin fibroblasts and chicken fibroblasts; each lot QC-tested by WB on these controls.

Eukaryotic cell lines

Policy information about cell lines and Sex and Gender in Research

Cell line source(s)	NIH 3T3 Swiss mouse embryo fibroblast cell line purchased from American Type Culture Collection (ATCC; CRL-1658)
Authentication	NIH 3T3 cell line was not independently authenticated as cells were utilised within 10 passages of acquisition from ATCC.
Mycoplasma contamination	NIH 3T3 cell line was not tested for mycoplasma contamination as cells were utilised within 10 passages of acquisition from ATCC.
Commonly misidentified lines (See ICLAC register)	N/A

Clinical data

Policy information about clinical studies

All manuscripts should comply with the ICMJE guidelines for publication of clinical research and a completed CONSORT checklist must be included with all submissions.

Clinical trial registration	NCT02408770
Study protocol	Study Protocol is provided in Supplementary Appendix 1
Data collection	Recruitment ran from 29/03/2016 to 11/03/2019 at The Nightingale Centre, Wythenshawe Hospital, Manchester UK. Data collection from these participants continued until the last participant had completed study procedures (July 2019). Stored biological samples continued to be analysed thereafter. The study was formally closed on 31/12/2023.
Outcomes	The primary endpoint was the change in epithelial cell proliferation measured by %Ki67 staining before and after treatment - see “Ki67 staining” section of Methods. Secondary endpoints were (1) percentage of luminal, basal and mixed colonies by morphological analysis of adherent feeder layer assay - see “2D Human mammary colony forming assay” section of Methods; (2) percentage of luminal progenitor cells (EPCAM+/CD49f+) by FACS analysis - see “Flow cytometry analysis” section of Methods; (3) tissue stiffness assessed as the reduced indentation modulus by atomic force microscopy - see “Tissue stiffness by atomic force microscopy” section of Methods; (4) mean tissue section percentage fibrillar collagen assessed by picrosirius red staining and polarised light microscopy - see “Picrosirius red staining and polarised light microscopy” section of Methods; (5) background parenchymal enhancement assessed by magnetic resonance imaging (MRI) - see “Magnetic resonance imaging (MRI)” section of Methods; (6) the side effect profile of UA in this patient population assessed by CTCAE v4.03.

Plants

Seed stocks	N/A
Novel plant genotypes	N/A
Authentication	N/A

Flow Cytometry

Plots

Confirm that:

☒ The axis labels state the marker and fluorochrome used (e.g. CD4-FITC).

☒ The axis scales are clearly visible. Include numbers along axes only for bottom left plot of group (a 'group' is an analysis of identical markers).

☒ All plots are contour plots with outliers or pseudocolor plots.

☒ A numerical value for number of cells or percentage (with statistics) is provided.

Methodology

Sample preparation	Normal breast tissue was minced into ~2mm³ fragments and incubated in a dissociation medium containing phenol red-free DMEM/F12, 25% BSA Fraction V, 1mg/mL collagenase/hyaluronidase, and 5µg/mL insulin. After overnight digestion at 37°C with shaking, the cell suspension was washed and centrifuged at 450 x g for 5 minutes at 4°C. The epithelial pellet was treated with 0.05% Trypsin-EDTA and 5 mg/mL dispase, resuspended in HBSS/Hepes/FBS, and filtered through 100µm and 40µm sieves to obtain a single cell suspension. Cells were counted and frozen in Bambanker freezing media. Cells from paired samples were then stained for flow cytometry using the following antibodies: CD31 biotin-conjugated (eBioscience, 13-0319-82), CD45 biotin-conjugated (BioLegend, 304004), APC-Cy7 streptavidin-conjugated (BioLegend, 405208), CD49f-APC (BioLegend, 313616) and EpCAM-FITC (StemCell Technologies, 10109).
Instrument	BD™ LSR II flow cytometer
Software	BD FACSDiva™ (v8.0)
Cell population abundance	N/A
Gating strategy	Following singlet, live/dead and lineage CD45+/CD31+ exclusion, mammary gland epithelial lineages were determined as Luminal Progenitor (LP, CD49f+/EPCAM+), Luminal Mature (LM, CD49f-/EPCAM+) or Basal (B, CD49f+/EPCAM-/lo).

☒ Tick this box to confirm that a figure exemplifying the gating strategy is provided in the Supplementary Information.
