# Supplementary material for: Anti-progestin therapy targets hallmarks of breast cancer risk
Source: Nature. 2025 Nov 5;648(8094):736–45. doi: 10.1038/s41586-025-09684-7 (PMC12711567; doi:10.1038/s41586-025-09684-7)
Supplement: Supplementary file 3 — Uncropped western blots from Figure 4b and Extended Data Figure 12a,b. [file 41586_2025_9684_MOESM3_ESM.pdf]

Supplementary Figure 1 - Uncropped western blots from Figure 4B and Extended Data Figure 12A-B

1989N + UA

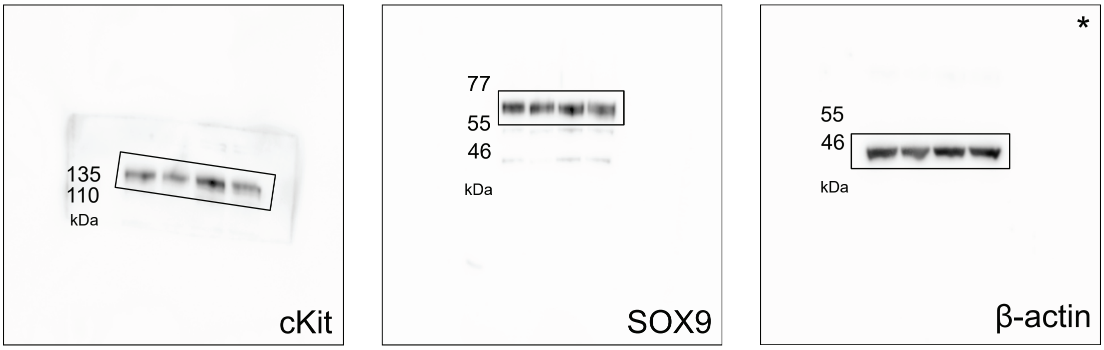

1989N + ON

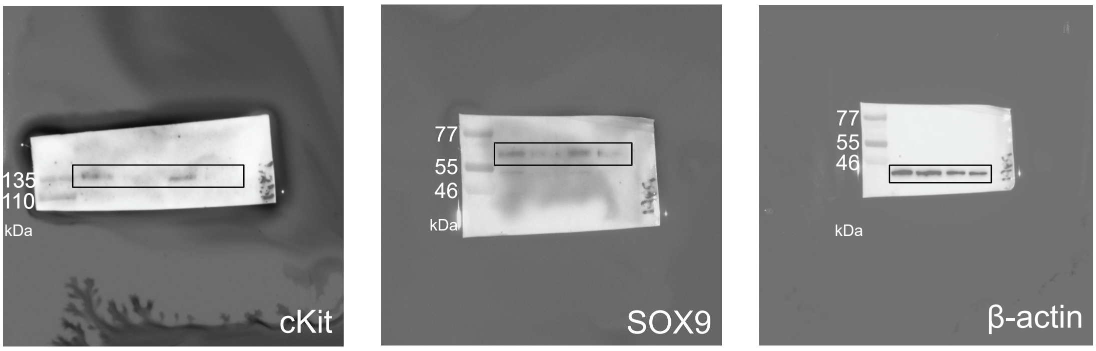

BB7162 + UA

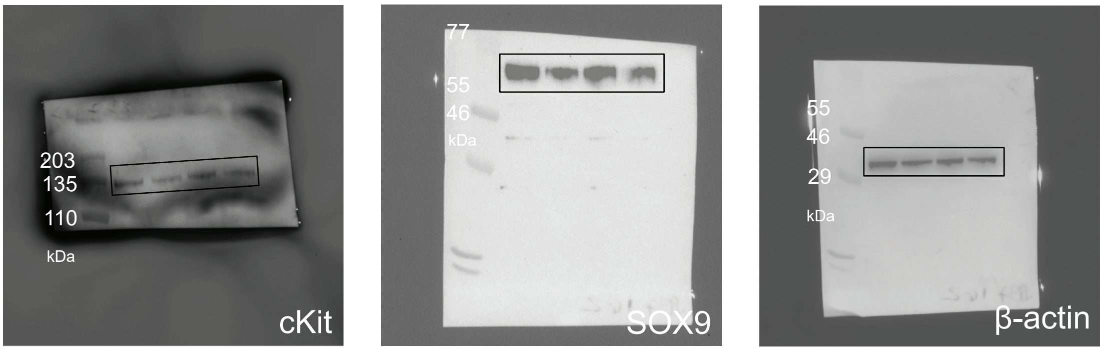

3088N + UA

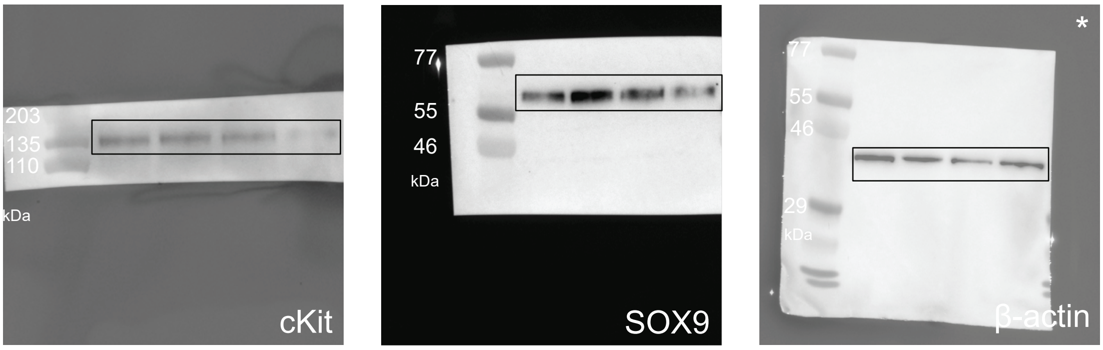

Supplementary Figure 1 (continuation)

BB7282 + ON

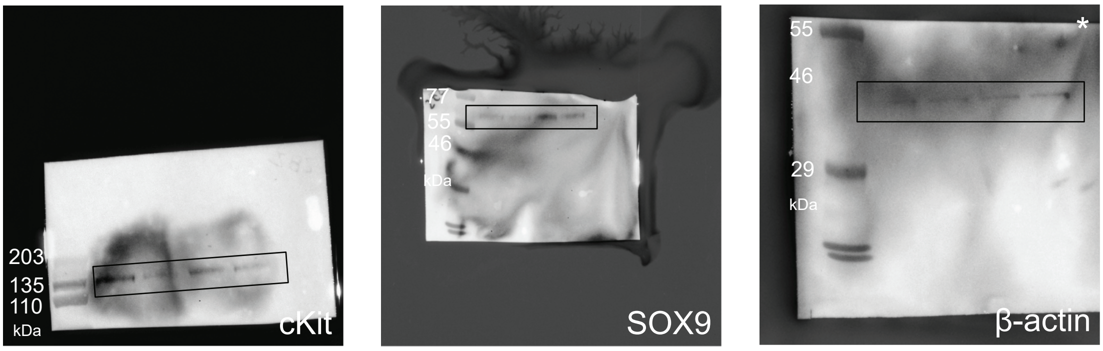

BB7073 + ON

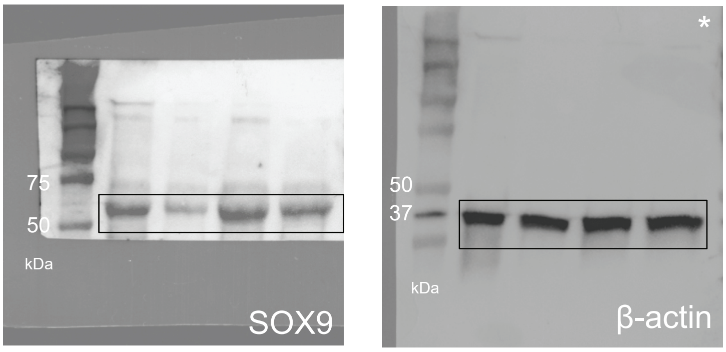

*β-actin was run on separate gels as sample processing control.

All gels/blots were processed in parallel.
