# Supplementary material for: Anti-progestin therapy targets hallmarks of breast cancer risk
Source: Nature. 2025 Nov 5;648(8094):736–45. doi: 10.1038/s41586-025-09684-7 (PMC12711567; doi:10.1038/s41586-025-09684-7)
Supplement: Supplementary file 4 — Supplementary Tables 1–8. [file 41586_2025_9684_MOESM4_ESM.zip › 2024-05-10498C-s4/Supplementary_Table_1.pdf]

Supplementary Table 1.

Study number	Lifetime BC Risk (%)	Age at consent	Age at FFTP	BMI	Density %VBD	VDG	Affected relatives	Probability BRCA1 PV (%)	Probability BRCA2 PV (%)	Bulk RnaSeq	scRNAseq	LCM Proteomics	Hyperion IMC	Paired MRI scans
01	30.6	42	32	26	10.6	3	M, 1MSDR, 2MTDR	0.22[a]	1.23[a]					✓
02	26.9	41	34	25	16.2	4	M, 1MSDR	0.22	0.51				✓	
03	33.8	39	29	28	10.8	3	M[bil]	2.41	4.13					✓
05	28.2	44	36	26	6.8	2	M (B+O), 1MSDR, 1PSDR	0.06	0.00[b]					✓
06	20.9	36	16	25	10.5	3	M[bil], 2MSDR	1.33[d]	1.37[d]			✓	✓	✓
07	17.0	43	24	30	8.5	3	M(O), 1MSDR	0.76[c]	0.18[c]					✓
10	27.3	40	32	25	10.6	3	M, 2PSDR(O), 1PTDR(O), 1MSDR,	0.45	0.00[b]	✓				✓
11	28.7	41	36	22	15	3	F, 1PSDR, 1MSDR	0.08[c]	1.26[c]					
12	23.3	36	29	22	15.7	4	M, 2MTDR	0.45[c]	0.24[c]	✓		✓	✓	✓
13	21.4	36	NK	25	9.2	3	M, 1MSDR(O)	0.06	0.00[b]					✓
14	28.5	39	27	25	8.6	3	M, 1MSDR	0.55	0.93				✓	✓
16	22.3	37	31	21	17.8	4	1MSDR[bil]	2	2.04					

17	28.8	39	nulip	24	8.6	3	M	0.38	4.33		✓	✓	✓	✓
18	38.3	36	NK	36	6.1	2	M, 1MSDR, 1PSDR, 1PTDR	4.2	4.19		✓			
19	27.1	36	31	28	20.7	4	M[bil], 1MSDR	0.61[a]	0.32[a]	✓				✓
21	22.5	44	35	30	6.3	2	2MSDR, 1MTDR[bil]	0.67	5.2					✓
22	24.1	35	nulip	33	5.7	2	M	0.36	0.62	✓			✓	
24	17.9	35	19	30	10.1	3	M, 1MTDR	0.76	1.09	✓	✓		✓	
25	21.6	41	25	29	17.9	4	M, 2MTDR	0.41	1.1	✓	✓			
26	23.7	39	nulip	42	2.0	1	2PSDR, 2PTDR	0.11	0.5	✓				
28	29.3	34	nulip	22	18.6	4	M, 2MSDR	0.37	0.8		✓			
30	20.4	39	27	28	5.4	2	3MSDR, 3MTDR	0.11	0.64	✓				
31	28.8	37	29	26	11	3	M, 1MSDR	0.25	0.64	✓	✓	✓	✓	
32	23.6	34	nulip	37	NK	NA	M	1	1.92	✓				
Median	25.5	39	29	26	10.5	-	-	0.43	1.39	-	-	-	-	-
N=	24	24	22	24	23	23	24	24	24	10	6	4	8	12

M mother; [bil] bilateral BC; F father; B+O breast and ovarian cancer; MSDR maternal second degree relative; PSDR paternal second degree relative; TDR third degree relative; [a] negative unaffected gene panel testing in participant; [b] affected first degree relative PV, direct gene test negative in participant (default mutation search sensitivities); [c] BRCA1/2 screen negative in affected FDR; [d] BRCA1/2 screen negative in affected second degree relative
