# Supplementary material for: Anti-progestin therapy targets hallmarks of breast cancer risk
Source: Nature. 2025 Nov 5;648(8094):736–45. doi: 10.1038/s41586-025-09684-7 (PMC12711567; doi:10.1038/s41586-025-09684-7)
Supplement: Supplementary file 4 — Supplementary Tables 1–8. [file 41586_2025_9684_MOESM4_ESM.zip › 2024-05-10498C-s4/Supplementary_Table_2.pdf]

Supplementary Table 2. All records of toxicity thought at least possibly related to UA by grade.

Symptomatic AE	Grade 1	Grade 2	Any Grade	%
breast pain/tenderness	10	1	11	42.3
headache	6	2	8	30.8
hot flushes	7	0	7	26.9
insomnia/sleep disturbance	5	0	5	19.2
nausea	5	0	5	19.2
agitation/irritability	4	0	4	15.4
dizziness/vertigo	4	0	4	15.4
abdominal pain/stomach cramps	4	0	4	15.4
rash – acneiform	3	0	3	11.5
confusion/lethargy	3	0	3	11.5
weight gain	3	0	3	11.5
urinary urgency	2	0	2	7.7
urinary incontinence	2	0	2	7.7
breast swelling	2	0	2	7.7
dry mouth	2	0	2	7.7
vaginal bleeding	2	0	2	7.7
somnolence	1	0	1	3.8
anxiety	1	0	1	3.8
presyncope	1	0	1	3.8
constipation	1	0	1	3.8
vaginal candida	1	0	1	3.8
bilateral knee swelling	1	0	1	3.8
hair thinning	1	0	1	3.8
pruritus	1	0	1	3.8
fatigue	1	0	1	3.8
vaginal discharge	1	0	1	3.8
vaginal dryness	1	0	1	3.8

Laboratory AE	Grade 1	Grade 2	Any Grade	%
raised ALT	3	0	3	11.5
reduced WCC	2	0	2	7.7
raised cholesterol	1	0	1	3.8
raised TSH	1	0	1	3.8
increased creatinine	1	0	1	3.8

VAB related AE*	Grade 1	Grade 2	Any Grade	%
bruising	26	0	26	100.0
pain	5	2	7	26.9
bleeding	2	1	3	11.5

* worst of 2 for each individual participant
