# Supplementary material for: Anti-progestin therapy targets hallmarks of breast cancer risk
Source: Nature. 2025 Nov 5;648(8094):736–45. doi: 10.1038/s41586-025-09684-7 (PMC12711567; doi:10.1038/s41586-025-09684-7)
Supplement: Supplementary file 4 — Supplementary Tables 1–8. [file 41586_2025_9684_MOESM4_ESM.zip › 2024-05-10498C-s4/Supplementary_Table_8.pdf]

Supplementary Table 8.

Biobank	Study ID	Lifetime Risk of BC (%)	Age at consent	Age at FFTP
MCRC	BB7073T1N	NK	67	20
MCRC	BB7133T1N	65	29	24
MCRC	BB7143T1N	NK	44	27
MCRC	BB7154T1N	50	46	40
MCRC	BB7158T1N	23	43	NK
MCRC	BB7162T1N	BRCA1 mutation carrier	26	NK
MCRC	BB7164T1N	33	49	36
MCRC	BB7282T1N	25	44	34
BCN	1989N	NK	21	NK
BCN	3088N	NK	24	NK
BCN	1715PM	NK (BRCA1/2 negative)	27	NK
BCN	1923PM	NK	45	Nulliparous
**Median**	NA	33	43.5	30.5
