# Supplementary material for: Anti-progestin therapy targets hallmarks of breast cancer risk
Source: Nature. 2025 Nov 5;648(8094):736–45. doi: 10.1038/s41586-025-09684-7 (PMC12711567; doi:10.1038/s41586-025-09684-7)
Supplement: Supplementary file 4 — Supplementary Tables 1–8. [file 41586_2025_9684_MOESM4_ESM.zip › 2024-05-10498C-s4/Supplementary-Table-legends.docx]

**Supplementary Table 1 -** Clinical and demographic data of BC-APPS1 study participants, with indication of samples included in OMICs analyses and MRI scans.

BC- breast cancer; FFTP - First Full-Term Pregnancy; BMI - Body Mass Index; VBD – Volumetric Breast Density; VDG - Volpara Density Grades; LCM - Laser Capture Microdissection; IMC - Imaging Mass Cytometry; MRI - Magnetic Resonance Imaging; NK- Not Known; NA – Not Applicable.

**Supplementary Table 2 -** All records of toxicity at least possibly related to UA, categorised by grade (CTCAE v4.0).

AE – Adverse Event; ALT - Alanine Transaminase; WCC - White Cell Count; TSH - Thyroid-Stimulating Hormone

**Supplementary Table 3 -** Number of cells in each of the seven broad cell types identified by scRNAseq from samples of six participants, shown at baseline and post-treatment timepoints.

**Supplementary Table 4 -** Full list of pairwise differentially expressed genes following UA treatment across the seven main cell types, as well as the subcluster populations FB1, FB2, FB3, and BMYO1. For each gene, the table reports p-values for differential expression (de_pval) and differential variance (dv_pval), along with the corresponding effect sizes: log fold change (de_coef) and variance coefficient (dv_coef).

**Supplementary Table 5 -** List of pairwise differentially expressed genes following UA treatment across the seven main cell types, subsetted to genes that encode proteins that act as ligands that bind to specific receptor proteins. For each gene, the table reports p-values for differential expression (de_pval) and differential variance (dv_pval), along with the corresponding effect sizes: log fold change (de_coef) and variance coefficient (dv_coef).

**Supplementary Table 6 -** List of pairwise differentially expressed genes following UA treatment across the seven main cell types, subsetted to extracellular matrix (ECM) genes through intersection with the “REACTOME Extracellular Matrix Organisation” gene set. For each gene, the table reports p-values for differential expression (de_pval) and differential variance (dv_pval), along with the corresponding effect sizes: log fold change (de_coef) and variance coefficient (dv_coef).

**Supplementary Table 7** - List of ligand–receptor pairs underlying significantly down-regulated collagen gene signalling interactions from basal and fibroblast cell states (sender cells) to all annotated breast cell states (receiver cells), as visualised in the chord diagrams of Figure 2K. For each interaction, the table lists the ligand and its source cell type, the corresponding receptor in the target cell population, and the computed probability of interaction (prob). The percentage of cells within the target population expressing the receptor (minimum 5% cutoff) is reported both at baseline and following UA treatment.

**Supplementary Table 8 -** Clinical information of Biobank patient dataset. For BCN Tissue Bank samples, patient IDs for normal cases are marked with an "N" while those from high-risk women undergoing prophylactic mastectomy are marked with a "PM" at the end of the ID.

BC- Breast Cancer; FFTP - First Full-Term Pregnancy; NK- Not Known; NA – Not Applicable.
